# No Evidence of Infrahumanization or Trait-Based Dehumanization in Children’s Evaluation of Gender Nonconforming Peers

**DOI:** 10.3390/bs16101743

**Published:** 2026-09-24

**Authors:** Marshall M. C. Hui, Karson T. F. Kung

**Affiliations:** Department of Psychology, University of Hong Kong, Hong Kong SAR, China; mhmc@connect.hku.hk

**Keywords:** dehumanization, infrahumanization, gender nonconformity, children, intergroup bias, prejudice, social cognition

## Abstract

A recent study (Hui & Kung, 2025), using a visual scale and a mind perception task, has found that children aged 9–12 years blatantly and subtly dehumanize gender nonconforming (GN) peers. While this recent study has provided some novel insights into children’s evaluation of GN peers, it remains unknown whether the observed effects can be generalized to other forms of dehumanization. In the broader dehumanization literature, some studies have reported that different dehumanization processes co-occur, but other studies have reported inconsistent findings across measures and forms of dehumanization. Extending Hui and Kung (2025), the present study examined two additional forms of dehumanization (infrahumanization and trait-based dehumanization) in 235 children aged 9–12 years. Using emotion and trait attribution tasks, children rated hypothetical gender conforming (GC) peers and GN peers on primary (or non-uniquely human) and secondary (or uniquely human) emotions as well as uniquely human (UH) and human nature (HN) traits. Children’s ratings were analyzed using mixed-design ANOVAs, with participant gender (boy, girl) and age group (9–10 y, 11–12 y) as between-subjects factors, and peer gender (boy, girl), peer gender conformity (GC, GN), and emotion/trait type (primary, secondary; UH, HN) as within-subjects factors. Results indicated that children did not differentially attribute secondary emotions or UH/HN traits to GC and GN peers. Contributing new developmental evidence based on a peer gender nonconformity paradigm, the present study augments the growing research literature suggesting that different forms of dehumanization are distinct processes that may not necessarily co-occur. The present study also concurs with recent critiques highlighting that some previously reported effects of infrahumanization and trait-based dehumanization may have been products of methodological artifacts confounding humanness with prosociality and valence in the trait stimuli employed.

## 1. Introduction

Gender nonconforming (GN) children are at heightened risk of bullying victimization and social ostracism compared to gender conforming (GC) children (Aspenlieder et al., 2009; Kwan et al., 2020; Toomey et al., 2014; Zosuls et al., 2016). One potential mechanism underlying such hostility towards GN peers is dehumanization. Applying dehumanization frameworks, Hui and Kung (2025) found that children aged 9–12 years rated GN peers as more animal-like and less human-like than GC peers (blatant dehumanization) and spontaneously attributed fewer mental states to GN peers than to GC peers (subtle dehumanization in the form of mind denial). In addition, in Hui and Kung (2025), children’s blatant dehumanization predicted bullying intentions towards GN peers. These findings suggest that, by middle childhood, children start to dehumanize GN peers which may contribute to children’s hostility towards GN peers.

Notably, dehumanization processes encompass multiple conceptualizations of humanness and distinct operationalizations (Fincher et al., 2018; Giner-Sorolla et al., 2021; Kteily & Landry, 2022; Landry & Seli, 2024), and dehumanization measures derived from different theoretical accounts do not always converge (Enock et al., 2021a, 2021b). Hui and Kung (2025) examined children’s GN-based dehumanization using only two dehumanization frameworks (i.e., blatant dehumanization and mind denial), leaving open the question of whether children also exhibit other forms of dehumanization of GN peers. The present study extends this work by applying two additional frameworks of dehumanization (i.e., infrahumanization and trait-based dehumanization) to examine children’s evaluation of GN peers.

### 1.1. Dehumanization as a Multifaceted Construct

Dehumanization refers to the socio-cognitive process of denying or overlooking aspects of a person’s humanity (Bandura, 2002; Haslam & Loughnan, 2014). It is a multifaceted construct encompassing distinct forms and processes, grounded in different conceptualizations and operationalizations of what it means to be human (Fincher et al., 2018; Giner-Sorolla et al., 2021; Kteily & Landry, 2022; Landry & Seli, 2024).

The related processes are often categorized into blatant or subtle dehumanization (Kteily & Landry, 2022). Blatant dehumanization involves explicitly likening others to lower-status animals or objects (Bai & Zhao, 2024; Kteily et al., 2015). Subtle dehumanization refers to the tendency to perceive others as possessing fewer mental states (i.e., mind denial; Epley & Waytz, 2010; Harris & Fiske, 2006), fewer human emotions (i.e., infrahumanization; Leyens et al., 2000, 2001), or fewer human traits (i.e., trait-based dehumanization or dual model of dehumanization; Haslam, 2006). Dehumanization can predict discrimination, aggression, and extreme violence towards stigmatized groups and individuals (Goff et al., 2008; Zlobina et al., 2023), even after controlling for prejudice (Bruneau et al., 2018; Kteily et al., 2015, 2016). Dehumanization may promote harm by disengaging moral restraints against harming others and by fostering indifference towards others’ suffering (Bandura, 2002; Harris & Delgado, 2025; Kteily & Landry, 2022).

#### 1.1.1. Infrahumanization

Among subtle dehumanization theories, one extensively studied framework is infrahumanization. According to the infrahumanization theory, emotions can be categorized into primary emotions (e.g., happiness, anger), which are perceived to be shared with animals, and secondary emotions (e.g., compassion, jealousy), which are perceived to be uniquely human (Demoulin et al., 2004; Haslam & Loughnan, 2014). The infrahumanization theory suggests that people often subtly dehumanize outgroups by attributing fewer secondary emotions to them than to ingroups, while attributing comparable levels of primary emotions to both ingroups and outgroups (Demoulin et al., 2004; Leyens et al., 2003, 2007). Infrahumanization is theorized to operate independently of prejudice; both positive and negative secondary emotions are attributed less to outgroups than to ingroups (Leyens et al., 2007; Rodríguez-Pérez & Betancor, 2023).

Infrahumanization is typically assessed using emotion attribution tasks, in which participants select emotion from a list (Leyens et al., 2001; Viki & Abrams, 2003) or rate emotion on a scale (Albarello & Rubini, 2012; Enock & Over, 2022; Enock et al., 2021b), evaluating whether different primary and secondary emotions are characteristic of different social groups. Other studies have assessed infrahumanization by asking participants to infer the occurrence or frequency of primary and secondary emotions experienced by a group (Baldry et al., 2015; Cuddy et al., 2007; Martin et al., 2008; Szuster & Wojnarowska, 2016; Wohl et al., 2012), and frequency of secondary emotion expression has also been used to manipulate infrahumanization in experimental paradigms (Martínez et al., 2017; Pereira et al., 2009). Prior studies with adults have reported evidence for infrahumanization in a wide range of intergroup contexts based on ethnicity (Gaunt, 2009; Leyens et al., 2001; Mosso & Russo, 2019), race (Albarello & Rubini, 2012; Banton et al., 2020), political orientation (Merola & McGlone, 2011), and gender (Rodrigues, 2025; Viki & Abrams, 2003). It has been reported that lower attribution of secondary emotions to outgroups predicts reduced willingness to help them (Baldry et al., 2015; Cuddy et al., 2007) and lower intentions to reconcile following intergroup conflicts (Tam et al., 2007; Wohl et al., 2012).

#### 1.1.2. Trait-Based Dehumanization

Trait-based dehumanization is another extensively studied framework of subtle dehumanization. Trait-based dehumanization is conceptualized as a process of denying others of traits that distinguish humans from animals and objects. According to this theory, there are two distinct dimensions of human traits: uniquely human (UH) traits, which distinguish humans from animals (e.g., rationality, civility), and human nature (HN) traits, which distinguish humans from machines or inanimate objects (e.g., warmth, cognitive openness) (Haslam, 2006). In theory, denying someone of UH traits results in animalistic dehumanization, whereby targets are perceived as animal-like (i.e., uncultured, irrational, amoral); denial of HN traits results in mechanistic dehumanization, whereby targets are perceived as machine-like (i.e., cold, rigid, passive) (Haslam, 2006; Haslam et al., 2007; Haslam & Loughnan, 2014). As with infrahumanization, trait-based dehumanization is also theorized to operate independently of prejudice (Haslam, 2006; Haslam & Stratemeyer, 2016).

Trait-based dehumanization is typically examined using trait attribution tasks, in which participants rate the extent to which they perceive social groups or targets to possess UH and HN traits. Prior studies with adults have reported evidence for trait-based dehumanization towards racial (Jardina & Piston, 2022), national (Bain et al., 2009), ethnic (Capozza et al., 2013; Vaes, 2023), political (Crawford et al., 2013), and gender/sexual minority outgroups (MacInnis & Hodson, 2012; Tanriverdi et al., 2024). It has been reported that lower attribution of human traits predicts greater punitive intentions, greater social exclusion, and reduced support and helping towards social groups (Andrighetto et al., 2014; Bastian & Haslam, 2011; Leidner et al., 2013; Terskova & Agadullina, 2019).

### 1.2. Dehumanization in Childhood

Although dehumanization has been extensively studied in adults, dehumanization processes in children remain underexplored (McLoughlin, 2023). Emerging evidence suggests that children also engage in blatant and subtle dehumanization. Children blatantly rate stigmatized groups as more animal-like and less human-like than non-stigmatized groups (Hui & Kung, 2025; Zhou & Hare, 2022). Children also spontaneously use fewer mental state words to describe stigmatized groups and outgroups than non-stigmatized groups and ingroups (Hui & Kung, 2025; McLoughlin & Over, 2017). Children’s blatant dehumanization predicts greater bullying and punitive intentions (Hui & Kung, 2025; Zhou & Hare, 2022, 2026), whereas findings on the behavioral consequences of mind perception or denial are mixed (Gönültaş et al., 2020; Hui & Kung, 2025; McLoughlin & Over, 2019; Reinecke et al., 2025).

Developmental studies have also examined infrahumanization and trait-based dehumanization in children. For example, in Brown et al. (2007), British children aged 11–16 years attributed fewer secondary emotions to students from another school than to students from their own school. In Martin et al. (2008), Scottish children aged 6–11 years expected their ingroup football team to experience more secondary than primary emotions, and expected an outgroup team to experience comparable levels of primary and secondary emotions. Similarly, in Chas et al. (2015), Spanish children aged 11–12 years attributed fewer secondary emotions to outgroup football fans than to ingroup fans. In Costello and Hodson (2014), White Canadian children aged 6–10 years attributed more secondary emotions and uniquely human traits to White peers than to Black peers. In van Noorden et al. (2014), Dutch children aged 7–12 years attributed fewer UH and HN traits to non-friends than to friends. In Fousiani et al. (2019), Cypriot adolescents in grades 10–11 attributed more UH and HN traits to ingroup bullies than to outgroup bullies. These findings on infrahumanization and trait-based dehumanization must be interpreted with caution in light of recent methodological critiques of dehumanization research.

### 1.3. Recent Debates and Methodological Concerns

Some researchers have noted that the design of many infrahumanization studies may have confounded humanness with prosociality in the emotion stimuli used (Bunce et al., 2025; Enock et al., 2021b; Over, 2021a, 2021b). Prosocial emotions are those that foster connection and promote reparative responses in relationships. According to Bunce et al. (2025), although many studies control for valence between primary and secondary emotions, 79.61% of the infrahumanization studies reviewed (121 out of 152 studies) used secondary emotions (e.g., remorse, guilt) that were more prosocial than primary emotions (e.g., anger, irritation). Consequently, lower attribution of secondary emotions to outgroups may reflect reduced attribution of prosocial emotions rather than uniquely human emotions. Echoing this concern, Enock et al. (2021b) found no evidence of infrahumanization when the prosociality of emotions was considered. In Enock et al. (2021b), participants did not attribute secondary emotions differently to ingroups and outgroups; instead, participants attributed fewer prosocial and more antisocial emotions to outgroups than to ingroups, regardless of whether the emotions were primary or secondary. Similarly, Bunce et al. (2025) tested different sets of emotion stimuli and found that infrahumanization effects emerged only when the stimuli confounded humanness with prosociality. Moreover, Enock and Over (2022) reported that secondary emotion attribution did not predict helping intentions after controlling for the prosociality of emotions.

Some researchers have also inquired about the validity of trait-based dehumanization findings, showing that many human trait measures used in prior studies have confounded humanness with valence (or desirability) (Brennan et al., 2024; Enock et al., 2021a; Over, 2021a, 2021b). Many trait-based dehumanization studies compute “humanness” composite scores using predominantly positive human traits (e.g., rational, kind-hearted; Capozza et al., 2013; Morris et al., 2018; Terskova & Agadullina, 2019), while overlooking negative traits that may also be perceived as uniquely human (e.g., arrogant, stingy; Enock et al., 2021a). In such cases, higher humanness scores indicate both greater humanness and greater positivity. Further complicating this issue, some studies compute “dehumanization” composite scores using primarily negative non-human traits (e.g., savage, immature, heartless; Bastian & Haslam, 2011; Kuljian & Hohman, 2023; Kunst et al., 2019; Pizzirani et al., 2019), neglecting potentially positive non-human traits (e.g., energetic, efficient; Enock et al., 2021a). Higher dehumanization scores then reflect both greater dehumanization and greater negativity. Consequently, group differences in human trait scores may reflect intergroup preference—attributing more positive traits to ingroups and more negative traits to outgroups—rather than dehumanization. Supporting this critique, Enock et al. (2021a) found no evidence of trait-based dehumanization when positive and negative human traits were equally represented. In Enock et al. (2021a), overall, participants did not attribute UH and HN traits differently to ingroups and outgroups; instead, they attributed more positive UH and HN traits to ingroups and more negative UH and HN traits to outgroups. Brennan et al. (2024) further reported that when both positive and negative human traits were included, attribution of human traits did not predict punitive intentions towards criminals.

Similar limitations exist in developmental research of infrahumanization and trait-based dehumanization. First, emotion and trait stimuli used in prior developmental studies often confounded humanness with valence and prosociality. For instance, secondary emotions tended to be more prosocial than primary emotions (Brown et al., 2007; Costello & Hodson, 2014; Martin et al., 2008), and human traits were often predominantly positive (Fousiani et al., 2019; van Noorden et al., 2014). Therefore, observed effects may partly reflect children’s tendency to attribute fewer prosocial emotions and fewer positive traits to outgroups, rather than children’s denial of human emotions and traits. Indeed, there is also evidence that when both positive and negative human traits were included in a study, children attributed fewer positive human traits and more negative human traits to non-friends than to friends (van Noorden et al., 2016). Second, most developmental studies relied on relatively small samples (Brown et al., 2007: *N* = 109; Chas et al., 2015: *N* = 48; Costello & Hodson, 2014: Study 1, *N* = 20; Study 2, *N* = 53; Martin et al., 2008: *N* = 90). The small samples may potentially inflate effect sizes and limit replicability of findings (Button et al., 2013). These methodological issues limit the inferences that can be drawn regarding the emergence of infrahumanization and trait-based dehumanization in childhood.

Beyond measurement concerns, some scholars have questioned the conceptual breadth and specificity of the dehumanization literature (Bloom, 2022; Over, 2021a, 2021b; Rai et al., 2018). It remains unclear to what extent the wide range of dehumanization operationalizations reflects a single underlying psychological construct or distinct processes within a multifaceted phenomenon (Kteily & Landry, 2022). Studies that employ and compare multiple dehumanization measures remain relatively scarce. Although some studies have reported that different dehumanization processes co-occur (Kim et al., 2025; Kteily et al., 2015), other studies have reported inconsistent findings across measures. For example, Enock et al. (2021a, 2021b) found that many outgroups, from minimal outgroups to criminals, were blatantly dehumanized but were not attributed fewer secondary emotions or fewer human traits than ingroups. Similarly, Kersbergen and Robinson (2019) found that Americans blatantly dehumanized obese people but did not infrahumanize them. In Sitruk et al. (2023), people with physical disabilities were blatantly dehumanized and attributed fewer mental states and human traits, but not fewer human emotions. Correlations between dehumanization measures range from null to moderate across studies, depending on the target, study design, and cultural context (Kersbergen & Robinson, 2019; Kteily et al., 2015; Sitruk et al., 2023). Different dehumanization processes may also predict distinct behavioral outcomes (Kteily et al., 2015; Waytz & Schroeder, 2014), and to varying degrees (Cheng et al., 2025), thereby requiring different intervention approaches to address them effectively (Zhou et al., 2024). Hence, effects observed based on one particular dehumanization framework or measure may not be generalizable to other forms of dehumanization.

The aforementioned critiques and findings do not necessarily invalidate the entire dehumanization literature, but they underscore the importance of employing more stringent methodological designs to examine different dehumanization processes in both child and adult samples. For instance, infrahumanization studies should use primary and secondary emotions matched for valence and prosociality, and trait-based dehumanization studies should include both positive and negative human traits. Moreover, comparing multiple forms of dehumanization processes would offer insights into which specific aspects of humanness are denied in social groups and whether dehumanization reflects a unified construct or a group of distinct, partially independent processes.

### 1.4. Present Study

Hui and Kung (2025) examined blatant and subtle dehumanization of GN peers in children across four age groups (5–6 years, 7–8 years, 9–10 years, and 11–12 years) using a visual scale and a mind perception task. It was found that, by age 9–10 years, children both blatantly rated GN peers as more animal-like/less human-like than GC peers and spontaneously attributed fewer mental state words to GN peers than GC peers. The present study extended Hui and Kung (2025) by examining whether children dehumanize GN peers via infrahumanization and trait-based dehumanization.

In the present study, using materials and procedures closely aligned with Hui and Kung (2025), children from two age groups (9–10 years and 11–12 years) in Hong Kong learned about four hypothetical peers who varied in peer gender (boy, girl) and peer gender conformity (GC, GN). Children rated these peers on primary and secondary emotions as well as UH and HN traits. The youngest age in the present study was 9 years, in line with the age when both blatant and mind denial forms of dehumanization of GN peers were observed in Hui and Kung (2025). In addition, some prior research has shown that children dehumanize outgroups via infrahumanization and trait-based dehumanization in middle and late childhood (Costello & Hodson, 2014; Martin et al., 2008; van Noorden et al., 2014), developmental periods that overlap with the age range (9–12 years) in the present study.

The present study addressed recent methodological concerns by employing emotion and trait words that are balanced for prosociality and valence, and by recruiting a sample larger than those in prior relevant developmental studies. Furthermore, to the authors’ best knowledge, existing studies on children’s infrahumanization and trait-based dehumanization have been conducted exclusively in Western cultural contexts. Although the present study did not have any specific cross-cultural hypotheses and cannot directly compare dehumanization processes across cultures, conducting research with children from a non-Western context can contribute to the diversity of the evidence base and the evaluation of the broader generalizability of developmental findings.

#### Prediction and Exploration

Across measures and outcomes, in the present study, participant gender (boy vs. girl) and participant age group (9–10 years vs. 11–12 years) were examined as between-subjects factors, and peer gender (boy vs. girl) and peer gender conformity (GC vs. GN) were examined as within-subjects factors. Two competing hypotheses were considered for the effects of peer gender conformity on children’s attribution of human emotions and traits. On the one hand, if different dehumanization accounts reflect a shared underlying mechanism, given that children blatantly dehumanize GN peers and subtly attribute reduced amounts of mental states to GN peers (Hui & Kung, 2025), they would also attribute fewer secondary emotions and fewer UH and/or HN traits to GN peers than to GC peers. Moreover, as prior studies have reported that adults attribute fewer secondary emotions and human traits to GN individuals (Rodrigues, 2025; Tanriverdi et al., 2024), similar patterns may be detected in childhood. On the other hand, if prior infrahumanization and trait-based dehumanization findings in adult and child samples were driven by methodological confounds as suggested by recent research (Bunce et al., 2025; Enock et al., 2021a, 2021b; Over, 2021a, 2021b), then children would not differentially attribute secondary emotions and human traits to GC and GN peers, as the measures employed took into account prosociality of secondary emotions and valence of UH and HN traits. The major competing hypotheses are as follows:

(a) *Infrahumanization*

**H_1a_.**  *Children attribute fewer secondary emotions to GN peers than to GC peers.*

**H_0a_.**  *Children attribute comparable secondary emotions to GN and GC peers.*

(b) *Trait-based dehumanization*

**H_1b_.**  *Children attribute fewer UH traits and/or fewer HN traits to GN peers than to GC peers.*

**H_0b_.**  *Children attribute comparable UH and HN traits to GN and GC peers.*

An exploratory approach was adopted to examine the potential moderation of participant age group, participant gender, and peer gender on GN-based dehumanization. Regarding participant age group, prior studies yielded inconsistent findings on the moderation of age group on dehumanization processes. While some studies found dehumanization to be relatively stable across age (Martin et al., 2008; van Noorden et al., 2014; Zhou & Hare, 2022), other studies found a developmental shift between age groups (Hui & Kung, 2025; McLoughlin & Over, 2017). Regarding participant gender, even though some studies found that boys exhibited stronger dehumanization towards others than did girls (Costello & Hodson, 2014; van Noorden et al., 2014), other studies did not find such effects based on participant gender (Martin et al., 2008; Hui & Kung, 2025). Regarding peer gender, as infrahumanization and trait-based dehumanization are often considered to arise from ingroup/outgroup differentiation (Leyens et al., 2000; Haslam & Loughnan, 2014), it is possible that children would attribute fewer secondary emotions and human traits to other-gender GN peers (double outgroup) than to own-gender GN peers (single outgroup). However, Hui and Kung (2025) found that children dehumanize both own-gender and other-gender GN peers via blatant dehumanization and mind denial to a similar degree. Given these mixed findings, there were no specific hypotheses concerning participant age group, participant gender, or peer gender.

## 2. Materials and Methods

### 2.1. Participants

A power analysis was conducted in G*Power 3.1 (Faul et al., 2009) for a mixed-design ANOVA examining within-between interactions. A minimum sample size of 236 participants was required to detect a small effect size (*f* = 0.10) with 90% power (α = 0.05, correlation among repeated measures = 0.30, nonsphericity correction ε = 1) given the multi-factorial mixed design. The correlation among repeated measures was specified as 0.30 rather than the G*Power’s default of 0.50, because a conservative choice was needed to hedge against underpowering if the true correlation was weaker than the default. This chosen value (0.30) is consistent with data from Hui and Kung (2025), which reported a mean within-subject correlation of *r* = 0.31.

A sample of 236 ethnically Chinese children in Hong Kong was recruited across two age groups: 9–10 years and 11–12 years. Each age group included 59 girls and 59 boys. One participant was excluded prior to analysis because the experimenter’s notes documented sustained off-task behaviors that compromised task administration. With the final sample of *N* = 235, achieved power to detect *f* = 0.10 under the specified parameters was 89.98%, indicating that the impact of exclusion on statistical power was negligible. Participants were recruited through a university, local primary schools, community centers, and online advertisements.

### 2.2. Materials

#### 2.2.1. Peer Vignettes

Peer vignettes, developed by Hui and Kung (2025), introduced participants to four hypothetical peers who varied in gender conformity and gender. These four hypothetical peers included a GC boy, a GN boy, a GC girl, and a GN girl. Each vignette included the following: (a) an avatar created using the Storyboard That website and (b) a written description specifying the peer’s gender and gender conformity. Peer gender was explicitly stated in the written description and further indicated by their names, hairstyles, school uniforms, and gender pronouns. Peer gender conformity was manipulated through gender-typed toy and playmate preferences, which show robust differences between boys and girls in prior meta-analytic and large-scale population research (Davis & Hines, 2020; Golombok et al., 2008; Iervolino et al., 2005; Kung, 2022; Kung et al., 2024). The GC boy and GN girl shared identical boy-typical preferences (i.e., toy gun, toy truck, playing with boys), whereas the GC girl and GN boy shared identical girl-typical preferences (i.e., doll, kitchen set, playing with girls). All peers were described as the same age and in the same grade as the participant. The peer vignettes are shown in Table S1 (see Supplementary Materials Section S1).

#### 2.2.2. Emotion Words

Four primary emotions (happiness, surprise, scare, sadness) and four secondary emotions (pride, hope, regret, guilt) were selected based on prior infrahumanization research (Demoulin et al., 2004; Martin et al., 2008; Vaes et al., 2003). In both adult and child samples, the selected secondary emotions have been rated as more uniquely human than the primary emotions (Demoulin et al., 2004; Enock et al., 2021b; Rodríguez et al., 2016; Rodríguez-Pérez et al., 2014). The number of positive and negative emotions was balanced within each of the primary and secondary emotion categories. An emotion stimuli selector developed by Bunce et al. (2025) was employed to statistically assess the perceived humanness, prosociality, and valence of emotion stimuli. Results indicated that the selected primary and secondary emotions differed significantly in perceived humanness (*p* < 0.001) but did not differ in prosociality or valence (*p*s > 0.05). All emotion words were included in the *Hong Kong Chinese Lexical Lists for Primary Learning* (HKSAR Education Bureau, 2007), indicating that they were age-appropriate and readily comprehensible for children in this age range.

#### 2.2.3. Trait Words

Four uniquely human (UH) traits (humble, polite, disorganized, stingy) and four human nature (HN) traits (creative, friendly, impulsive, impatient) were selected based on prior trait-based dehumanization research (Bastian & Haslam, 2011; Enock et al., 2021a; Kteily et al., 2015). These traits have been shown to load highly and uniquely onto their respective humanness dimensions (Enock et al., 2021a; Haslam et al., 2005). To address concerns about confounding humanness with trait valence, an equal number of positive and negative traits was included on both UH and HN measures. Again, all of these trait words were included in the *Hong Kong Chinese Lexical Lists for Primary Learning* (HKSAR Education Bureau, 2007), indicating that they were age-appropriate and readily comprehensible for children in this age range.

#### 2.2.4. Composite Construction and Internal Consistency

For the main statistical analyses reported subsequently in the Results Section, each composite (i.e., primary emotions, secondary emotions, UH traits, HN traits) combined positive- and negative-valence items. The main analyses were based on four-item composites combining items across valence.

For internal consistency analyses, a different approach was taken, using separate two-item positive-valence and two-item negative-valence subscales (e.g., positive secondary emotions, negative secondary emotions). A different approach was needed for internal consistency, because covariance among items in composites combining positive- and negative-valence items would likely be driven downward by the mixed-valence measure design. In this context, the use of Cronbach’s *α* based on mixed-valence composites may not be appropriate. Indeed, prior infrahumanization and trait-based dehumanization studies employing mixed-valence stimuli have generally not reported internal consistency indices for their humanness composites (e.g., Brennan et al., 2025; Enock et al., 2021a; Enock & Over, 2023). To address this mixed-valence issue, internal consistency of items in the present study was evaluated separately by valence for each composite. Instead of using Cronbach’s α, which underestimates reliability of short subscales (Cortina, 1993; Eisinga et al., 2013), the Spearman–Brown coefficient was computed following the recommendation of Eisinga et al. (2013), alongside the inter-item correlation, which serves as an interpretable index of scale homogeneity (Clark & Watson, 1995). In the present study, inter-item correlations ranged from 0.34 to 0.54, falling within or above the recommended range of 0.15–0.50 (Clark & Watson, 1995), and Spearman–Brown coefficients ranged from 0.51 to 0.70. Detailed reliability statistics are reported in Supplementary Materials Section S4.

### 2.3. Procedures

Participants were assessed individually in a quiet room at their school, a community center, or the researchers’ university. The testing session lasted approximately 30–40 min. Experimenters included the first author and trained psychology undergraduate research helpers. Experimenters administered the procedures based on a standardized experimental protocol developed prior to the start of data collection. Upon completion, participants received a small gift (e.g., stationery) and a participation certificate as compensation.

#### 2.3.1. Peer Vignette Manipulation

After a brief warm-up, each participant was presented with the four peer vignettes (GC boy, GN boy, GC girl, GN girl) one at a time in an order counterbalanced across participants. The experimenter read each vignette aloud. For example, the GN girl vignette was introduced as follows: “*She is Siu Ting (a girl-typical name). She is a girl. She is your age and in your grade. She likes playing with toy guns and toy trucks. She likes playing with boys. People think she is boyish.*” After each vignette, participants were asked three attention-check questions assessing their understanding of the peer’s gender conformity: (1) “*Does [peer’s name] like playing with toy guns and toy trucks or kitchen sets and dolls?*”; (2) “*Does [peer’s name] like playing with boys or girls?*”; (3) “*Do people think [peer’s name] is boyish or girlish?*”. If a participant answered any question incorrectly, the same vignette was shown and read again. No participant failed the attention checks twice for any peer.

Participants then completed two measures: an emotion attribution scale and a trait attribution scale. The order of the two scales was counterbalanced across participants. Task order did not alter the conclusions of the planned analyses (see Supplementary Materials Section S8).

#### 2.3.2. Emotion Attribution Scale

Participants were instructed to rate how frequently they think each peer experienced different emotions in daily life: “*We all experience different emotions. I want you to rate how often you think these children experience the following emotions in their everyday life.*” Following certain prior infrahumanziation research (Martin et al., 2008; Szuster & Wojnarowska, 2016), participants were asked to rate the frequency with which each peer experienced primary and secondary emotions, rather than how characteristic these emotions were of each peer. As children construe emotions as temporary experiential states (Kramer et al., 2024; Pons et al., 2004), frequency ratings are more developmentally appropriate and comprehensible for children than are characteristic-based judgements used in some adult infrahumanization studies. The experimenter read each emotion aloud. Standardized definitions were provided upon request (see Table S2 in Supplementary Materials Section S1). After instructions, participants responded on their own using a 4-point Likert scale presented via Qualtrics: 0 = never, 1 = rarely, 2 = sometimes, 3 = always. Visual bars of increasing length accompanied response options to facilitate comprehension of the gradation of options. While rating each peer, icons of the peer and his/her preferred toys were displayed at the top of the screen as visual reminders. Participants were reminded that there were no right or wrong answers before rating. To reduce experimenter and social desirability biases, the experimenter looked away while participants were rating. Each participant completed the scale for each peer one by one, in the same order as the peer vignette presentation for the participant. Mean scores (0–3) were computed separately for primary and secondary emotions for each hypothetical peer.

#### 2.3.3. Trait Attribution Scale

Participants were instructed to attribute UH and HN traits to each hypothetical peer: “*We all have different traits and qualities. I want you to rate whether these children have the following traits and qualities.*” Trait words were read aloud by the experimenter. Standardized definitions were provided upon request (see Table S3 in Supplementary Materials Section S1). Participants responded on a 4-point Likert scale: 0 = not at all, 1 = a tiny bit, 2 = quite a bit, 3 = very much. Visual gradation bars were again presented. Participants were reminded that there were no right or wrong answers before rating. To reduce experimenter and social desirability biases, the experimenter looked away while participants were rating. Each participant completed the scale for each peer one by one, in the same order as the peer vignette presentation for the participant. Mean scores (0–3) were computed for UH and HN traits for each hypothetical peer.

### 2.4. Analytic Plan

All analyses were conducted in SPSS version 31.0.2.0. Prior infrahumanization and trait-based dehumanization research has predominantly employed ANOVA-based analyses (Brennan et al., 2024; Enock et al., 2021a, 2021b; Haslam et al., 2005; Leyens et al., 2001). The present study built on Hui and Kung (2025), which also employed ANOVA-based analyses. Following prior studies, and given the mixed between- and within-subjects structure of the present design, mixed-design ANOVA was adopted as the primary analytic approach. Linear mixed-effects models are additionally reported in Supplementary Materials Section S3 as a robustness check.

Separate mixed-design ANOVAs were conducted for emotion and trait ratings. For both analyses, participant gender (boy vs. girl) and age group (9–10 years vs. 11–12 years) were included as between-subjects factors, while peer gender (boy vs. girl) and peer gender conformity (GC vs. GN) were included as within-subjects factors. For emotion ratings, emotion type (primary vs. secondary) was included as a within-subjects factor. For trait ratings, trait type (UH vs. HN) was included as a within-subjects factor. Significant interactions were followed up with planned simple interactions and simple effects analyses.

In the present study, following the analytic approach adopted in Hui and Kung (2025), all pairwise follow-up tests were conducted using the Fisher’s Least Significant Difference (LSD) procedure. Employing the same correction approach for pairwise follow-up tests in the present study can ensure consistency in statistical evaluation criteria across studies testing different dehumanization frameworks (i.e., avoid using more lenient or more stringent criteria for certain frameworks).

Effect sizes were evaluated using partial eta squared ($\eta_{p}^{2}$) for ANOVAs and Cohen’s *d* for pairwise comparisons. Cohen’s *d*s for between-subjects contrasts were computed using the pooled standard deviation. Cohen’s *d*s for within-subjects contrasts were computed using the average of the two within-condition standard deviations.

Normality was examined using skewness and kurtosis statistics, both of which indicated no substantial deviations from normality (all values within acceptable range between −1.5 and +1.5; Tabachnick & Fidell, 2019). Levene’s tests were non-significant for all within-subjects cells (*p*s > 0.05), except for HN trait ratings of GC boys (*p* = 0.010). ANOVA is suggested to be robust to variance heterogeneity when between-subjects cell sizes are balanced (Blanca et al., 2018), as is the case with the present research design. Sphericity was automatically satisfied because all within-subjects factors comprised only two levels.

As previously reviewed, infrahumanization and trait-based dehumanization are theorized to operate independently of intergroup preferences (Haslam, 2006; Leyens et al., 2000). Following the analytical approach employed by prior studies (Bain et al., 2009; Kteily et al., 2015; Leyens et al., 2001; Loughnan et al., 2009), emotion valence and trait valence were not included as factors in the primary analyses. For completeness, additional analyses that included emotion valence and trait valence as factors were conducted and are reported in Supplementary Materials Section S5. These additional analyses did not alter the pattern or interpretation of the primary analyses.

The present study was not preregistered. All stimuli and materials are provided in Supplementary Materials. Data availability is described in the Data Availability Statement.

## 3. Results

Descriptive statistics are presented in Table S4 in Section S2 of Supplementary Materials. Observed cell-level means ranged from 1.28 to 1.85 for emotion ratings and from 1.43 to 1.80 for trait ratings, clustering around the scale midpoint (1.50) rather than at either endpoint, indicating that responses were not subject to floor or ceiling effects.

### 3.1. Emotion Ratings

The main effect of age group was significant, *F*(1, 231) = 10.26, *p* = 0.002, $\eta_{p}^{2}$ = 0.04. Emotion ratings given by participants in the 11–12 age group (*M* = 1.61, *SD* = 0.32) were significantly higher than those by participants in the 9–10 age group (*M* = 1.48, *SD* = 0.27). The age group × participant gender interaction was significant, *F*(1, 231) = 5.77, *p* = 0.017, $\eta_{p}^{2}$ = 0.02. Post hoc analyses were conducted separately for participant gender. Emotion ratings among boys in the 11–12 age group (*M* = 1.67, *SD* = 0.36) were significantly higher than those among boys in the 9–10 age group (*M* = 1.45, *SD* = 0.29), *p* < 0.001, *d* = 0.65, whereas emotion ratings among girls in the 9–10 age group (*M* = 1.51, *SD* = 0.25) and 11–12 age group (*M* = 1.54, *SD* = 0.26) did not significantly differ, *p* = 0.571, *d* = 0.12.

The main effect of emotion type was significant, *F*(1, 231) = 191.15, *p* < 0.001, $\eta_{p}^{2}$ = 0.45. Ratings of primary emotions (*M* = 1.71, *SD* = 0.29) were significantly higher than those of secondary emotions (*M* = 1.39, *SD* = 0.40). The peer gender × peer gender conformity interaction was also significant, *F*(1, 231) = 7.56, *p* = 0.006, $\eta_{p}^{2}$ = 0.03. Post hoc analyses were conducted separately for each peer gender. Emotion ratings for GN boys (*M* = 1.56, *SD* = 0.39) were significantly higher than those for GC boys (*M* = 1.51, *SD* = 0.35), *p* = 0.033, *d* = 0.14, whereas emotion ratings for GC girls (*M* = 1.59, *SD* = 0.37) were significantly higher than those for GN girls (*M* = 1.54, *SD* = 0.36), *p* = 0.025, *d* = 0.14.

The lower-order effects above were qualified by a significant peer gender × peer gender conformity × emotion type interaction, *F*(1, 231) = 14.45, *p* < 0.001, $\eta_{p}^{2}$ = 0.06; the simple effects reported above are presented for completeness, and interpretation should be based on the decomposition of the three-way interaction as follows. Post hoc analyses were conducted separately for each emotion type. For primary emotions, the peer gender × peer gender conformity interaction was significant, *F*(1, 231) = 22.10, *p* < 0.001, $\eta_{p}^{2}$ = 0.09. Ratings of primary emotions were significantly higher for GN boys (*M* = 1.75, *SD* = 0.41) than for GC boys (*M* = 1.63, *SD* = 0.41), *p* < 0.001, *d* = 0.28. In contrast, ratings of primary emotions were significantly higher for GC girls (*M* = 1.78, *SD* = 0.43) than for GN girls (*M* = 1.66, *SD* = 0.39), *p* < 0.001, *d* = 0.28. For secondary emotions, the peer gender × peer gender conformity interaction was not significant, *F*(1, 231) = 0.35, *p* = 0.557, $\eta_{p}^{2}$ = 0.00. Participants did not attribute secondary emotions differently across peer gender or peer gender conformity conditions (all *p*s > 0.05). Specifically, ratings of secondary emotions for GN boys (*M* = 1.36, *SD* = 0.53) did not significantly differ from those for GC boys (*M* = 1.38, *SD* = 0.49), *p* = 0.628, *d* = 0.03, and ratings of secondary emotions for GN girls (*M* = 1.41, *SD* = 0.52) did not significantly differ from those for GC girls (*M* = 1.39, *SD* = 0.48), *p* = 0.655, *d* = 0.03. All other main effects and interactions were not significant (see Table S5 in Supplementary Materials Section S2). Figure 1 presents the mean of emotion ratings across peer gender, peer gender conformity, and emotion type.

To evaluate the null finding, a Bayesian directional paired-samples *t*-test was conducted on the focal contrast between GC and GN peers for secondary emotions. The analysis yielded strong evidence that children attributed secondary emotions comparably to GC and GN peers, *BF*_0−_ = 13.11 (see Supplementary Materials Section S6). To check whether or not this null pattern was attributable to difficulty comprehending the emotion words, a sensitivity analysis excluding participants who requested clarification for any emotion word was conducted. This sensitivity analysis yielded the same pattern of results, further confirming the null findings (see Supplementary Materials Section S7).

### 3.2. Trait Ratings

The main effect of age group was significant, *F*(1, 231) = 8.09, *p* = 0.005, $\eta_{p}^{2}$ = 0.03. Trait ratings given by participants in the 11–12 age group (*M* = 1.66, *SD* = 0.21) were significantly higher than those by participants in the 9–10 age group (*M* = 1.58, *SD* = 0.23). The main effect of trait type was significant, *F*(1, 231) = 24.14, *p* < 0.001, $\eta_{p}^{2}$ = 0.10. Ratings of HN traits (*M* = 1.66, *SD* = 0.25) were significantly higher than ratings of UH traits (*M* = 1.58, *SD* = 0.27). The main effect of peer gender conformity was non-significant, *F*(1, 231) = 0.78, *p* = 0.377, $\eta_{p}^{2}$ = 0.00. Participants did not attribute human traits differently to GC peers (*M* = 1.62, *SD* = 0.25) and GN peers (*M* = 1.63, *SD* = 0.25). The peer gender × peer gender conformity interaction was non-significant, *F*(1, 231) = 2.62, *p* = 0.107, $\eta_{p}^{2}$ = 0.01. The interaction between peer gender conformity and trait type was also not significant, *F*(1, 231) = 0.61, *p* = 0.437, $\eta_{p}^{2}$ = 0.00. Specifically, participants did not attribute UH traits differently to GC peers (*M* = 1.57, *SD* = 0.29) and to GN peers (*M* = 1.59, *SD* = 0.32), *p* = 0.217, *d* = 0.07; participants also did not attribute HN traits differently to GC peers (*M* = 1.66, *SD* = 0.30) and to GN peers (*M* = 1.67, *SD* = 0.29), *p* = 0.926, *d* = 0.01. All other main effects and interactions were not significant (see Table S6 in Supplementary Materials). Figure 2 presents the mean of trait ratings across peer gender, peer gender conformity, and trait type.

To evaluate the null findings, Bayesian directional paired-samples *t*-tests were conducted on the focal contrasts between GC and GN peers for aggregated human traits and separately for UH and HN traits. All three analyses yielded strong evidence that children attributed human traits comparably to GC and GN peers, *BF*_0−_ = 24.55 for aggregated human traits, *BF*_0−_ = 29.12 for UH traits, and *BF*_0−_ = 14.84 for HN traits (see Supplementary Materials Section S6). To check whether or not this null pattern was attributable to difficulty comprehending the trait words, a sensitivity analysis excluding participants who requested clarification for any trait word was conducted. This sensitivity analysis yielded the same pattern of results, further confirming the null findings (see Supplementary Materials Section S7).

## 4. Discussion

According to prior work by Hui and Kung (2025), children dehumanize gender nonconforming (GN) peers through blatant dehumanization and subtle mind denial—rating GN peers as more animal-like/less human-like than gender conforming (GC) peers and spontaneously attributing fewer mental states to GN peers than to GC peers. The present study extended these prior findings by examining whether children would also dehumanize GN peers through infrahumanization and trait-based dehumanization. Specifically, this study tested whether children would attribute fewer secondary emotions and fewer uniquely human (UH) and human nature (HN) traits to GN peers than to GC peers. Contrary to these theoretical predictions, as shown in statistical significance testing, children did not differentially attribute secondary emotions or UH/HN traits to GC and GN peers. In terms of effect size magnitude, the differences between GC and GN peers in secondary emotion attribution (*d* = 0.03), UH trait attribution (*d* = 0.07), and HN trait attribution (*d* = 0.01) were all negligible, falling well below the conventional threshold for a small effect (Cohen, 1988). Taken together, these findings suggest no infrahumanization or trait-based dehumanization in children’s evaluation of GN peers.

The current findings differ from some prior findings suggesting that children attribute fewer human emotions and traits to outgroups and stigmatized groups (Brown et al., 2007; Chas et al., 2015; Martin et al., 2008; van Noorden et al., 2014). In the present study, children did not attribute fewer human emotions or traits to GN peers than to GC peers. Children also did not attribute fewer human emotions or traits to gender outgroups than to gender ingroups; boys did not attribute fewer human emotions or traits to girl peers, and girls did not attribute fewer human emotions or traits to boy peers.

### 4.1. Potential Explanations

One possible explanation for this discrepancy concerns the methodological limitations of prior research. Previous developmental studies of infrahumanization and trait-based dehumanization have often used emotion and trait words that confound humanness with valence and prosociality (Bunce et al., 2025; Enock et al., 2021a, 2021b; Over, 2021a, 2021b). Thus, some previously reported positive findings detecting infrahumanization and trait-based dehumanization in young children (as young as age 6 years) may partly reflect early-emerging intergroup preferences (Dunham et al., 2008; Qian et al., 2019) that were inadvertently captured by the confounded measures. The present study adopted a more stringent test by using emotion stimuli that differed in humanness but were matched on valence and prosociality, and by including both positive and negative human traits. As indicated by the current findings, when these confounds were addressed, there was no evidence of infrahumanization or trait-based dehumanization towards GN peers or gender outgroups. Future developmental research using similarly balanced designs may examine whether the current findings can be generalized to other social targets.

Another possible explanation is that children in middle childhood may not yet have developed the capacities underlying infrahumanization and trait-based dehumanization processes. Although the developmental trajectories of different dehumanization processes remain underexplored, some studies suggest that blatant dehumanization and subtle mind denial may develop alongside children’s mentalizing capacities in early and middle childhood (McLoughlin & Over, 2017; Zhou & Hare, 2026), potentially emerging earlier than infrahumanization and trait-based dehumanization that require abstract conceptual understanding of humanness (Hui & Kung, 2025; Khanjanian et al., 2026; McLoughlin, 2023). While children in middle childhood can distinguish uniquely human from non-uniquely human emotions and traits (Rodríguez et al., 2016; Chas et al., 2015) and attribute human characteristics differently between humans and animals/robots (Manzi et al., 2020; Neldner et al., 2026), applying these distinctions to differentiate subgroups within the same human category may require more advanced reasoning abilities and greater knowledge of socially constructed group stereotypes (Rodríguez-Pérez et al., 2021). Individuals may associate gender nonconformity with other stigmatized identities (e.g., LGBTQ+, Henry & Steiger, 2022; Howansky et al., 2020) and accumulate stereotypes about GN individuals by observing maltreatment directed at them (Aspenlieder et al., 2009; Fasoli et al., 2016). It is possible that these stigma association and stereotyping may not be strong enough for related processes such as infrahumanization and trait-based dehumanization to emerge until late childhood or adolescence. Future research may examine whether adolescents attribute fewer secondary emotions and human traits to GN peers than to GC peers.

A third possible explanation is that Chinese children may not exhibit the same pattern of infrahumanization and trait-based dehumanization documented in Western children (Brown et al., 2007; Chas et al., 2015; Martin et al., 2008; van Noorden et al., 2014). Although the primary–secondary emotion and UH–HN trait distinctions are recognized across cultures (Demoulin et al., 2004; Haslam et al., 2008), research with adults has shown that humanness dimensions vary in their salience across cultural contexts (Bain et al., 2012; Vaes et al., 2012) and that people from distinct cultural backgrounds dehumanize particular social targets to varying degrees and along different humanness dimensions (Bain et al., 2009; Bruneau et al., 2018). It is possible that developmental trajectories of infrahumanization and trait-based dehumanization differ across Chinese and Western cultures. However, unlike the present study, prior studies with children in Western settings documenting effects of infrahumanization or trait-based dehumanization were based on confounded stimuli. In other words, the present study differed from prior studies in terms of both cultural setting and stimuli construction. Hence, empirically, existing evidence would not allow any clear adjudication between the cultural account and the methodological account. Future developmental research studies using balanced human emotion or trait stimuli in Western samples would help clarify the relevant cultural and methodological issues.

A related cultural mechanism concerns gender norms perception. Crucially, prior studies have reported that Chinese children express greater hostility towards GN peers than do children in other cultures (Kwan et al., 2024; Wang et al., 2022) and that gender nonconformity is a particularly salient social category shaping Chinese children’s peer preferences (Qian et al., 2021). Therefore, one would likely expect particularly strong infrahumanization and trait-based dehumanization of GN peers among Chinese children, a prediction that is not supported by the current null findings.

Detecting effects unrelated to dehumanization, the current analyses revealed that children attributed more primary emotions to GN boys than to GC boys, and to GC girls than to GN girls; that is, children attributed more primary emotions to peers with feminine interests than to peers with masculine interests. This pattern is consistent with well-documented gender stereotypes portraying feminine individuals as more emotionally expressive (Fischer & LaFrance, 2015; Kring & Gordon, 1998). As gender (non)conformity in the present study was operationalized through peers’ play preferences, this interaction effect on primary emotions may reflect stereotype inferences cued by these feminine versus masculine activity interests. This association aligns with recent evidence that activity-based cues are particularly consequential for children’s social reasoning about gender nonconforming peers (Schroeder & Fulcher, 2025). Importantly, this pattern in relation to feminine interests was confined to primary emotions and did not extend to secondary emotions, UH traits, or HN traits, the categories that are contingent to testing infrahumanization and trait-based dehumanization. The observed effects on primary emotions therefore reflect gender-emotionality stereotypes rather than dehumanization of peers with feminine interests.

### 4.2. Theoretical and Practical Implications

More broadly, the current findings have theoretical implications for the dehumanization literature. Collectively, findings from Hui and Kung (2025) and the present study support the view that dehumanization is a multifaceted construct comprising distinct processes of humanness denial (Landry & Seli, 2024). Prior research indicates that different operationalizations of dehumanization do not always converge (Kersbergen & Robinson, 2019; Kteily et al., 2015; Sitruk et al., 2023). In the context of children’s evaluation of GN peers, there is evidence of blatant dehumanization and subtle dehumanization in the form of mind denial (Hui & Kung, 2025), but there is no evidence of infrahumanization or trait-based dehumanization. This divergence suggests that blatant dehumanization and mind denial may be developmentally and psychologically distinct from infrahumanization and trait-based dehumanization. Blatant dehumanization and mind denial may reflect broader, more global judgments of diminished humanness that emerge relatively early (Hui & Kung, 2025; Zhou & Hare, 2022, 2026) and may not necessarily depend on social appraisals of human emotions or traits (Brennan et al., 2025; Enock et al., 2021a, 2021b). Indeed, blatant dehumanization and subtle mind denial can be driven by other factors such as low group status (Bruneau et al., 2018; Harris & Fiske, 2006), norm violation (Chen-Xia et al., 2024; Moreno-Gata et al., 2025), unfamiliarity (Harris & Fiske, 2011), emotional cost of interaction (Harris & Delgado, 2025), perceived threat (Gönültaş et al., 2020) and disgust (Harris & Fiske, 2006; Landry et al., 2022). Taken together, prior and current findings illustrate the importance of adopting multi-method approaches to investigate the specific ways social targets are dehumanized and the conditions under which different forms of dehumanization converge. Building on this multi-method perspective, to address on-going theoretical debates, future dehumanization research may apply structural equation modeling to statistically test whether different dehumanization measures reflect a shared latent structure or dissociable constructs.

The present study also has practical implications for developing interventions to improve children’s evaluation and behavioral treatment of GN peers. Different dehumanization processes may require different intervention approaches (Zhou et al., 2024). Given that 9- to 12-year-old children dehumanize GN peers through blatant dehumanization and mind denial (Hui & Kung, 2025), but not through infrahumanization or trait-based dehumanization, intervention approaches involving mentalization (McLoughlin & Over, 2019), intergroup contact (Bruneau et al., 2021), and counteracting negative misperceptions (Bruneau et al., 2022; Landry et al., 2023) would likely be more useful than interventions highlighting human traits and emotions in GN peers (Capozza et al., 2017; Enock & Over, 2022; Vaes et al., 2002). In applied settings, anti-bullying initiatives may prioritize activities that promote recognition of GN peers’ mental experiences (e.g., thoughts, feelings, goals), foster contact with GN peers and counteract misperceptions of GN peers, rather than emphasizing GN peers’ human traits and emotions. The current findings may inform targeted pre-service and in-service teacher training and inclusive school policies aimed at cultivating gender-inclusive educational practices and environments that support the wellbeing of GN children (Meyer & Leonardi, 2018; Omercajic & Martino, 2020). Nonetheless, empirical research testing interventions targeting children’s dehumanization processes remains limited and has often yielded null findings (Costello & Hodson, 2014; Zhou et al., 2024). Future research may test and compare whether these different intervention strategies can reduce children’s dehumanization of and hostility towards GN peers.

### 4.3. Limitations

The present study is not without limitations. Although the primary and secondary emotion stimuli in the present study were balanced in terms of prosociality, both categories primarily consisted of prosocial emotions. Some scholars have suggested that infrahumanization research could include both prosocial and antisocial primary and secondary emotions as stimuli in order to compare the influence of intergroup preference and dehumanization on emotion attributions and downstream behavioral outcomes (Enock & Over, 2022; Enock et al., 2021b). This approach is valuable. However, the primary goal of the present study was not to compare dehumanization and intergroup preference, but to test whether children would infrahumanize GN peers when emotion valence and prosociality are taken into account. Moreover, antisocial secondary emotions (e.g., contempt, schadenfreude) identified in prior research may be developmentally inappropriate or conceptually too complex for children in this age range.

Another issue is that the present study used a limited number of words to assess children’s emotion and trait attributions. There is no consensus on the optimal number of items for testing human emotion and trait attribution. Prior dehumanization research has varied widely in the number of items used to measure each emotion or trait category (i.e., primary emotions, secondary emotions, UH traits, HN traits), ranging from more than eight items per category (Crawford et al., 2013; Kteily et al., 2015; MacInnis & Hodson, 2012) to as few as one or two items per category (Demoulin et al., 2005; Vaes et al., 2003). Consistent with prior developmental research on dehumanization (Costello & Hodson, 2014; Martin et al., 2008; van Noorden et al., 2014), the present study used four words per category. Given that every child in the present study was asked to rate multiple hypothetical peers, including more items would have substantially lengthened the session and increased cognitive demands. However, a larger item pool per emotion or trait category would enable more precise reliability estimates of human emotion and trait composites. Future research may replicate and extend the current findings by using a broader range of emotion and trait stimuli.

While the present study adds to the field by contributing developmental evidence from a non-Western setting, the current measures and findings may be limited by potential cross-cultural differences. To reduce demands for child participants and to avoid lengthy testing sessions, the present study did not include a manipulation check to verify that Chinese children perceived the primary/secondary emotions and UH/HN traits along the intended humanness and valence dimensions. The present study drew stimuli from existing, validated sets used in earlier dehumanization studies with adults and children (Bastian & Haslam, 2011; Demoulin et al., 2004; Enock et al., 2021a, 2021b; Haslam et al., 2005; Kteily et al., 2015; Rodríguez et al., 2016; Rodríguez-Pérez et al., 2014). These sets and norms were established predominantly in Western and adult samples, and equivalent research resources for Chinese child participants are not available. Although the overall relevant humanness perception may not necessarily substantially differ across cultures or age groups, two potential concerns should be noted. First, some prior research with Spanish-speaking samples suggests that adults and children show broadly convergent primary/secondary emotion classifications but may differ in the degree to which they perceive specific terms as uniquely human (Rodríguez et al., 2016; Rodríguez-Pérez et al., 2014). Second, certain stimulus words may carry culturally distinct connotations in Chinese-speaking populations. For instance, *humility* is often considered a strongly socialized virtue in Chinese culture (Ying et al., 2024), which may shift the word’s valence and affect the valence matching within the UH composite. Taken together, developmental and/or cultural differences in how Chinese children conceptualize the humanness and valence of emotions and traits (McLoughlin, 2023; Vaes et al., 2012) may have contributed to the present null findings. Further research may collect humanness and valence judgments directly from Chinese children for measure development.

In contrast with some prior studies that used a characteristic-based response format to measure infrahumanization (Leyens et al., 2001; Viki & Abrams, 2003), the present study used a frequency-based response format (Baldry et al., 2015; Cuddy et al., 2007; Martin et al., 2008; Szuster & Wojnarowska, 2016; Wohl et al., 2012). Frequency-based responses may, in theory, be shaped by inferences about a target’s situational opportunities to experience an emotion, in addition to attributions about the target’s emotional capacity. Further research may directly compare the use of frequency-based and characteristic-based response formats in assessing infrahumanization to test if effects vary according to response format.

Although participants rated peers in the emotion and trait attribution tasks independently and were explicitly told that there were no correct or wrong answers, the presence of an adult experimenter in the room might still have introduced some social desirability pressure. This could attenuate differential attribution of emotions and traits to peers. The present findings should therefore be interpreted with caution.

The present study was conducted in a single Asian cultural context with children aged 9–12 years, constraining the generalizability of the current findings. More broadly, prior developmental studies of children’s dehumanization vary substantially in terms of age range and cultural context (Brown et al., 2007; Chas et al., 2015; Martin et al., 2008; van Noorden et al., 2014). This sample heterogeneity may contribute to the mixed findings in the literature. Additional developmental studies based on independent, diverse samples may shed light on the degree of replicability, which would likely depend on cultural context and age. Furthermore, prior research with adults has shown that culture and cultural orientation may play a role in shaping dehumanization (Bain et al., 2009; Kim et al., 2025). Future developmental research would benefit from investigating whether and how cultural orientation at individual, group, and societal levels may moderate children’s dehumanization.

Finally, the present study specifically examined peer gender and peer gender nonconformity but cannot provide insights into broader intersectionality issues. Prior research has examined how peer race, gender, and gender nonconformity may jointly affect children’s friendship preferences, showing that preferences are more strongly driven by peer gender and gender nonconformity than by race (Qian et al., 2021). Moreover, research has documented that gender nonconformity is elevated in neurodivergent children and youth compared with their neurotypical counterparts (e.g., Au Yeung et al., 2026; To et al., 2026; To & Kung, 2025), and the extent to which neurodivergent individuals are dehumanized has been shown to be associated with the extent to which they are perceived as gender nonconforming (Elderkin & Todd, 2026). Future studies may employ increasingly intersectional paradigms that include additional group memberships or characteristics to examine how different dehumanization processes interact across social contexts.

## 5. Conclusions

The present study examined children’s infrahumanization and trait-based dehumanization towards GN peers using a set of relatively balanced emotion and trait stimuli. Children aged 9 to 12 years did not attribute human emotions or traits differently to GN and GC peers, although children in this age group have previously been found to show blatant dehumanization and mind denial of GN peers. There is no evidence that children dehumanize GN peers via infrahumanization or trait-based dehumanization. Further research may compare the developmental trajectories of different dehumanization processes.

## Figures and Tables

**Figure 1:**
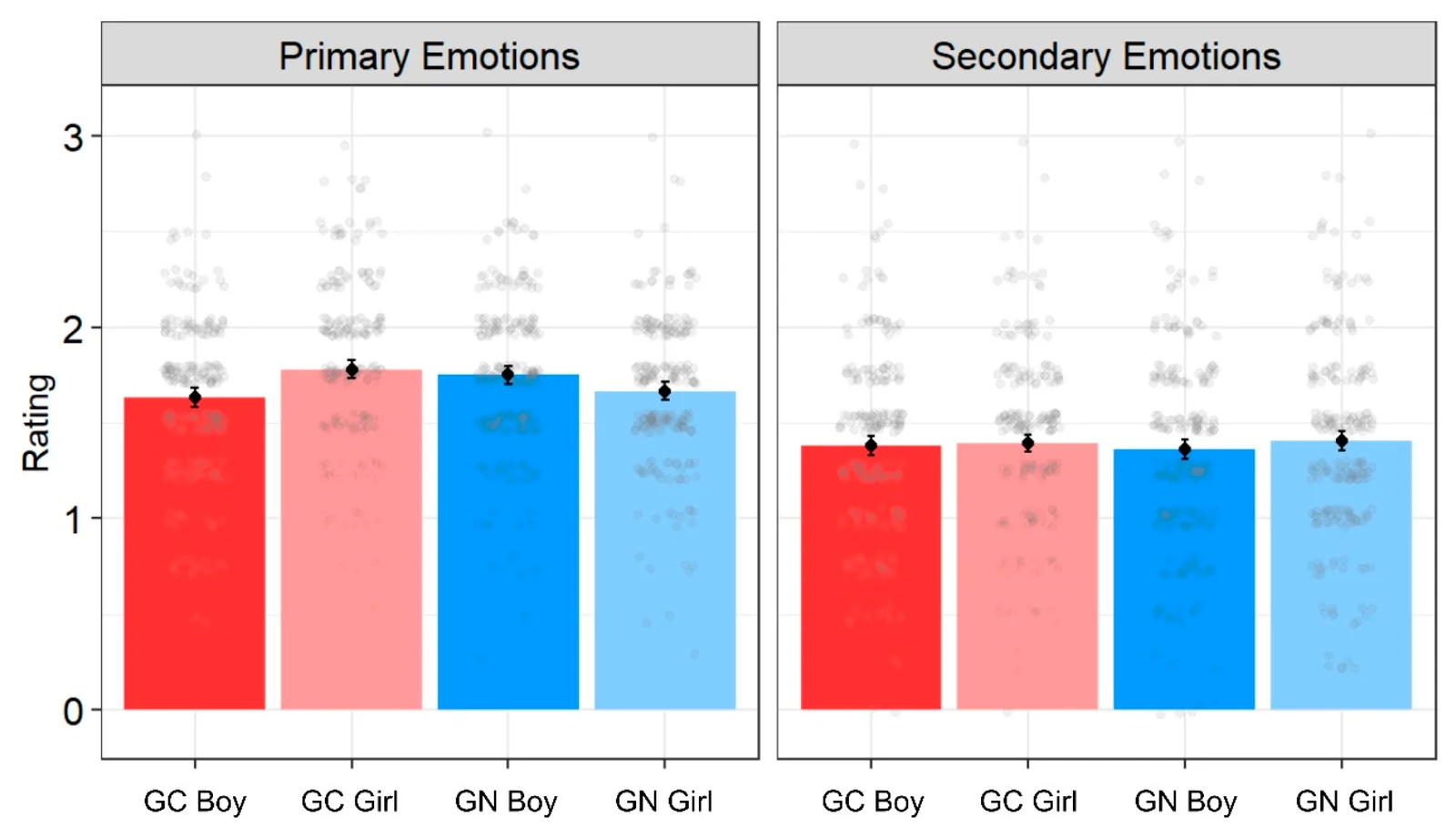
Mean emotion ratings as a function of peer gender, peer gender conformity, and emotion type. GC = gender conforming; GN = gender nonconforming. Error bars represent 95% confidence intervals.

**Figure 2:**
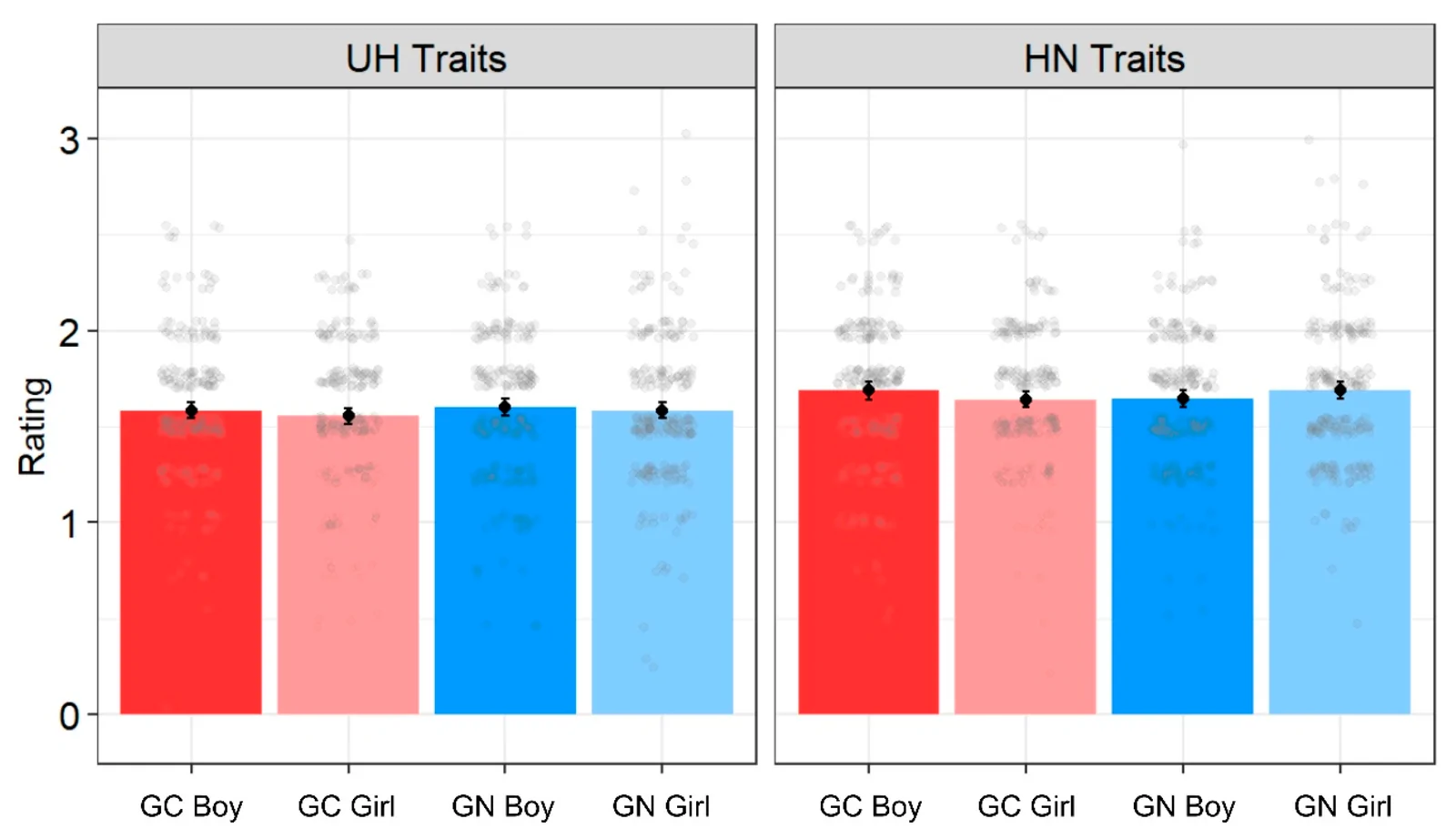
Mean trait ratings as a function of peer gender, peer gender conformity, and trait type. UH—uniquely human; HN—human nature; GC—gender conforming; GN—gender nonconforming. Error bars represent 95% confidence intervals.

## Data Availability

The data presented in this study are available from the corresponding author upon reasonable request. The data are not publicly available due to confidentiality and research ethics issues.

## Supplementary Materials

The following supporting information can be downloaded at: https://www.mdpi.com/article/10.3390/bs16101743/s1, Table S1: Peer vignette samples; Table S2: Standardized descriptions of emotion words; Table S3: Standardized descriptions of trait words; Table S4: Means and standard deviations of emotion and trait ratings by condition; Table S5: Full ANOVA results for emotion ratings; Table S6: Full ANOVA results for trait ratings; Table S7: Linear mixed-effects model for emotion ratings; Table S8: Linear mixed-effects model for trait ratings; Table S9: Inter-item correlations and Spearman–Brown reliability coefficients for 2-item emotion subscales, by peer gender, peer gender conformity, emotion type, and emotion valence; Table S10: Inter-item correlations and Spearman–Brown reliability coefficients for 2-item trait subscales, by peer gender, peer gender conformity, trait type, and trait valence; Table S11: Within- vs. between-emotion-type mean inter-item correlations, by peer gender, peer gender conformity, and emotion valence; Table S12: Within- vs. between-trait-type mean inter-item correlations, by peer gender, peer gender conformity, and trait valence; Table S13: ANOVA results for emotion ratings, including emotion valence as a within-subjects factor; Table S14: ANOVA results for trait ratings, including trait valence as a within-subjects factor; Table S15: ANOVA results for emotion ratings, including task order as a between-subjects factor; Table S16: ANOVA results for trait ratings, including task order as a between-subjects factor; Figure S1: Mean emotion ratings as a function of peer gender, peer gender conformity, emotion type, and emotion valence; Figure S2: Mean trait ratings as a function of peer gender, peer gender conformity, trait type, and trait valence; Figure S3: Robustness checks for Bayesian analyses of GC-GN contrasts in secondary emotions ratings; Figure S4: Robustness checks for Bayesian analyses of GC-GN contrasts in human trait aggregate, UH trait, and HN trait ratings.

## Abbreviations

The following abbreviations are used in this manuscript:

GC	Gender conforming
GN	Gender nonconforming
UH	Uniquely human
HN	Human nature
