# Supplementary material for: No Evidence of Infrahumanization or Trait-Based Dehumanization in Children’s Evaluation of Gender Nonconforming Peers
Source: Behav Sci (Basel). 2026 Sep 24;16(10):1743. doi: 10.3390/bs16101743 (PMC13701279; doi:10.3390/bs16101743)
Supplement: Supplementary file 1 [file behavsci-16-01743-s001.zip › behavsci-4438285-supplementary.pdf]

SUPPLEMENTARY MATERIALS

Section S1: Detailed Stimuli

Table S1. Peer vignette samples

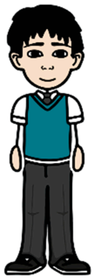 他是小俊，是一個男孩子。<br>他和你一樣大，讀同一年級。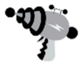<br>他喜歡玩玩具槍和玩具車。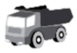<br>他喜歡和男孩子一起玩。<br>他讓人覺得像一個男孩子。	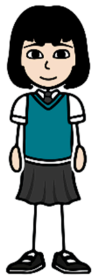 她是小美，是一個女孩子。<br>她和你一樣大，讀同一年級。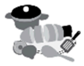<br>她喜歡玩煮飯仔和洋娃娃。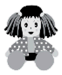<br>她喜歡和女孩子一起玩。<br>她讓人覺得像一個女孩子。
**Gender conforming (GC) boy vignette sample**<br>English translation:<br>He is Siu Chun (a boy-typical name).<br>He is a boy.<br>He is your age and in your grade.<br>He likes playing with toy guns and toy trucks.<br>He likes playing with boys.<br>People think he is boyish.	**Gender conforming (GC) girl vignette sample**<br>English translation:<br>She is Siu Mei (a girl-typical name).<br>She is a girl.<br>She is your age and in your grade.<br>She likes playing with kitchen sets and dolls.<br>She likes playing with girls.<br>People think she is girlish.
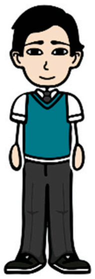 他是小軒，是一個男孩子。<br>他和你一樣大，讀同一年級。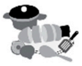<br>他喜歡玩煮飯仔和洋娃娃。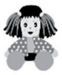<br>他喜歡和女孩子一起玩。<br>他讓人覺得像一個女孩子。	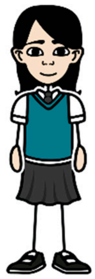 她是小婷，是一個女孩子。<br>她和你一樣大，讀同一年級。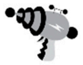<br>她喜歡玩玩具槍和玩具車。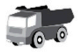<br>她喜歡和男孩子一起玩。<br>她讓人覺得像一個男孩子。
**Gender nonconforming (GN) boy vignette sample**<br>English translation:<br>He is Siu Hin (a boy-typical name).<br>He is a boy.<br>He is your age and in your grade.<br>He likes playing with kitchen sets and dolls.<br>He likes playing with girls.<br>People think he is girlish.	**Gender nonconforming (GN) girl vignette sample**<br>English translation:<br>She is Siu Ting (a girl-typical name).<br>She is a girl.<br>She is your age and in your grade.<br>She likes playing with toy guns and toy trucks.<br>She likes playing with boys.<br>People think she is boyish.

Note. The pairing of different avatars and GC/GN characteristics was counterbalanced across participants (i.e., each boy or girl avatar was GC for half of the participants and GN for the other half).

Table S2. Standardized descriptions of emotion words

Emotion Type	Emotion Valence	Word	Description
Primary	Positive	Happiness (開心)	Feeling joyful and in a good mood
		Surprise (驚喜)	Feeling amazed when something happens suddenly
	Negative	Scare (害怕)	Feeling very afraid
		Sadness (傷心)	Feeling unhappy and down
Secondary	Positive	Hope (有希望)	Feeling like there is a good chance of success
		Pride (自豪)	Feeling pleased and satisfied after doing something well
	Negative	Regret (後悔)	After doing something, wishing you had done it differently
		Guilt (內疚)	Feeling bad about yourself because you hurt someone or did something wrong

Table S3. Standardized descriptions of trait words

Trait Type	Trait Valence	Word	Description
Uniquely Human (UH)	Positive	Humble (謙虛)	Willing to learn from others, not always telling people how great you are
		Polite (有禮貌)	Showing respect to others and having good manners
	Negative	Disorganized (不整齊)	Leaving things around carelessly and making everything look untidy
		Stingy (小氣)	Not willing to share what you have with others
Human Nature (HN)	Positive	Creative (有創意)	Having lots of new ideas and thinking of things that others may not think of
		Friendly (友善)	Being kind to others and getting along well with them
	Negative	Impulsive (衝動)	Often doing things without staying calm or thinking about what might happen afterward
		Impatient (無耐性)	Not good at waiting, and always wanting things to happen right away

Section S2: Descriptive Statistics and Full ANOVA Results

Table S4. Means and standard deviations of emotion and trait ratings by condition

Participant Gender	Age Group	Peer Gender	Peer Gender Conformity	Emotion Ratings		Trait Ratings	
				Primary	Secondary	Uniquely Human	Human Nature
				M (*SD*)	*M* (*SD*)	*M* (*SD*)	*M* (*SD*)
Boy	9–10y	Boy	GC	1.52 (0.43)	1.32 (0.51)	1.47 (0.36)	1.66 (0.48)
			GN	1.67 (0.47)	1.29 (0.61)	1.52 (0.40)	1.63 (0.41)
		Girl	GC	1.66 (0.45)	1.28 (0.46)	1.43 (0.44)	1.64 (0.41)
			GN	1.58 (0.34)	1.33 (0.52)	1.54 (0.45)	1.57 (0.37)
	11–12y	Boy	GC	1.78 (0.45)	1.51 (0.57)	1.66 (0.35)	1.76 (0.32)
			GN	1.83 (0.39)	1.55 (0.55)	1.68 (0.36)	1.64 (0.34)
		Girl	GC	1.83 (0.45)	1.53 (0.51)	1.62 (0.35)	1.61 (0.32)
			GN	1.79 (0.42)	1.56 (0.51)	1.63 (0.41)	1.72 (0.42)
Girl	9–10y	Boy	GC	1.65 (0.37)	1.31 (0.43)	1.59 (0.40)	1.67 (0.40)
			GN	1.76 (0.37)	1.33 (0.48)	1.59 (0.44)	1.61 (0.41)
		Girl	GC	1.79 (0.37)	1.34 (0.43)	1.55 (0.36)	1.61 (0.36)
			GN	1.62 (0.44)	1.29 (0.49)	1.54 (0.41)	1.66 (0.34)
	11–12y	Boy	GC	1.57 (0.36)	1.37 (0.42)	1.61 (0.35)	1.66 (0.40)
			GN	1.74 (0.38)	1.28 (0.45)	1.61 (0.34)	1.68 (0.32)
		Girl	GC	1.85 (0.43)	1.42 (0.47)	1.62 (0.36)	1.70 (0.33)
			GN	1.67 (0.35)	1.45 (0.52)	1.63 (0.33)	1.80 (0.40)

Note. GC = Gender conforming; GN = Gender nonconforming

Table S5. Full ANOVA results for emotion ratings

Variables	*F*	*p*	η_p^2
**Age Group**	**10.26**	**.002**	**.04**
Participant Gender	0.77	.380	.00
**Age Group × Participant Gender**	**5.77**	**.017**	**.02**
Peer Gender	3.82	.052	.02
Peer Gender × Age Group	3.15	.077	.01
Peer Gender × Participant Gender	1.85	.175	.01
Peer Gender × Age Group × Participant Gender	3.56	.061	.02
Peer Gender Conformity	0.00	.990	.00
Peer Gender Conformity × Age Group	0.00	.948	.00
Peer Gender Conformity × Participant Gender	2.36	.126	.01
Peer Gender Conformity × Age Group × Participant Gender	0.02	.887	.00
**Emotion Type**	**191.15**	**< .001**	**.45**
Emotion Type × Age Group	0.96	.329	.00
Emotion Type × Participant Gender	2.20	.140	.01
Emotion Type × Age Group × Participant Gender	0.12	.731	.00
**Peer Gender × Peer Gender Conformity**	**7.56**	**.006**	**.03**
Peer Gender × Peer Gender Conformity × Age Group	0.36	.547	.00
Peer Gender × Peer Gender Conformity × Participant Gender	1.43	.233	.01
Peer Gender × Peer Gender Conformity × Age Group × Participant Gender	0.07	.789	.00
Peer Gender × Emotion Type	0.01	.932	.00
Peer Gender × Emotion Type × Age Group	0.12	.727	.00
Peer Gender × Emotion Type × Participant Gender	0.01	.932	.00
Peer Gender × Emotion Type × Age Group × Participant Gender	0.02	.880	.00
Peer Gender Conformity × Emotion Type	0.00	.952	.00
Peer Gender Conformity × Emotion Type × Age Group	0.02	.893	.00
Peer Gender Conformity × Emotion Type × Participant Gender	0.06	.810	.00
Peer Gender Conformity × Emotion Type × Age Group × Participant Gender	0.66	.418	.00
**Peer Gender × Peer Gender Conformity × Emotion Type**	**14.45**	**< .001**	**.06**
Peer Gender × Peer Gender Conformity × Emotion Type × Age Group	0.01	.929	.00
Peer Gender × Peer Gender Conformity × Emotion Type × Participant Gender	1.03	.311	.00
Peer Gender × Peer Gender Conformity × Emotion Type × Age Group × Participant Gender	3.33	.069	.01

Table S6. Full ANOVA results for trait ratings

Variables	*F*	*p*	η_p^2
**Age Group**	**8.09**	**.005**	**.03**
Participant Gender	0.55	.459	.00
Age Group × Participant Gender	0.69	.406	.00
Peer Gender	0.48	.490	.00
Peer Gender × Age Group	0.86	.356	.00
Peer Gender × Participant Gender	1.85	.175	.01
Peer Gender × Age Group × Participant Gender	2.19	.140	.01
Peer Gender Conformity	0.78	.377	.00
Peer Gender Conformity × Age Group	0.25	.617	.00
Peer Gender Conformity × Participant Gender	0.01	.929	.00
Peer Gender Conformity × Age Group × Participant Gender	0.60	.440	.00
**Trait Type**	**24.14**	**< .001**	**.10**
Trait Type × Age Group	0.96	.329	.00
Trait Type × Participant Gender	0.01	.937	.00
Trait Type × Age Group × Participant Gender	2.96	.087	.01
Peer Gender × Peer Gender Conformity	2.62	.107	.01
Peer Gender × Peer Gender Conformity × Age Group	0.56	.455	.00
Peer Gender × Peer Gender Conformity × Participant Gender	0.08	.776	.00
Peer Gender × Peer Gender Conformity × Age Group × Participant Gender	0.78	.379	.00
Peer Gender × Trait Type	0.52	.472	.00
Peer Gender × Trait Type × Age Group	0.20	.656	.00
Peer Gender × Trait Type × Participant Gender	1.10	.296	.01
Peer Gender × Trait Type × Age Group × Participant Gender	0.01	.927	.00
Peer Gender Conformity × Trait Type	0.61	.437	.00
Peer Gender Conformity × Trait Type × Age Group	2.30	.131	.01
Peer Gender Conformity × Trait Type × Participant Gender	3.48	.063	.02
Peer Gender Conformity × Trait Type × Age Group × Participant Gender	0.34	.560	.00
Peer Gender × Peer Gender Conformity × Trait Type	1.64	.202	.01
Peer Gender × Peer Gender Conformity × Trait Type × Age Group	1.50	.222	.01
Peer Gender × Peer Gender Conformity × Trait Type × Participant Gender	0.03	.871	.00
Peer Gender × Peer Gender Conformity × Trait Type × Age Group × Participant Gender	2.91	.089	.01

Section S3: Linear Mixed-Effects Model Analyses

Following a recommendation by an anonymous reviewer, supplementary linear mixed-effects model analyses with random intercept were conducted for emotion ratings and trait ratings in SPSS version 31.0.2.0.

Table S7. Linear mixed-effects model for emotion ratings

Variables	*F*	*p*
**Age Group**	**10.26**	**.002**
Participant Gender	0.77	.380
**Age Group × Participant Gender**	**5.77**	**.017**
Peer Gender	3.07	.080
Peer Gender × Age Group	2.54	.111
Peer Gender × Participant Gender	1.49	.222
Peer Gender × Age Group × Participant Gender	2.86	.091
Peer Gender Conformity	0.00	.992
Peer Gender Conformity × Age Group	0.00	.958
Peer Gender Conformity × Participant Gender	1.51	.219
Peer Gender Conformity × Age Group × Participant Gender	0.01	.909
**Emotion Type**	**353.47**	**< .001**
Emotion Type × Age Group	1.77	.184
**Emotion Type × Participant Gender**	**4.07**	**.044**
Emotion Type × Age Group × Participant Gender	0.22	.640
**Peer Gender × Peer Gender Conformity**	**8.62**	**.003**
Peer Gender × Peer Gender Conformity × Age Group	0.41	.520
Peer Gender × Peer Gender Conformity × Participant Gender	1.63	.202
Peer Gender × Peer Gender Conformity × Age Group × Participant Gender	0.08	.775
Peer Gender × Emotion Type	0.01	.936
Peer Gender × Emotion Type × Age Group	0.11	.743
Peer Gender × Emotion Type × Participant Gender	0.01	.936
Peer Gender × Emotion Type × Age Group × Participant Gender	0.02	.887
Peer Gender Conformity × Emotion Type	0.00	.961
Peer Gender Conformity × Emotion Type × Age Group	0.01	.912
Peer Gender Conformity × Emotion Type × Participant Gender	0.04	.843
Peer Gender Conformity × Emotion Type × Age Group × Participant Gender	0.45	.504
**Peer Gender × Peer Gender Conformity × Emotion Type**	**14.55**	**< .001**
Peer Gender × Peer Gender Conformity × Emotion Type × Age Group	0.01	.929
Peer Gender × Peer Gender Conformity × Emotion Type × Participant Gender	1.04	.309
Peer Gender × Peer Gender Conformity × Emotion Type × Age Group × Participant Gender	3.35	.067

Consistent with the original mixed-design ANOVA, the main effect of age group, $F(1, 231) = 10.26$, $p = .002$, the age group × participant gender interaction, $F(1, 231) = 5.77$, $p = .017$, the main effect of emotion type, $F(1, 1617) = 353.47$, $p < .001$, the peer gender × peer gender conformity interaction, $F(1, 1617) = 8.62$, $p = .003$, and the peer gender × peer gender conformity × emotion type interaction, $F(1, 1617) = 14.55$, $p < .001$, were significant.

In the linear mixed-effects models, an additional emotion type × participant gender interaction emerged, $F(1, 1617) = 4.07$, $p = .044$. Post hoc simple effects analyses revealed that primary emotion ratings among boys ($M = 1.71$, $SD = 0.34$) and girls ($M = 1.71$, $SD = 0.24$) did not significantly differ, $p = .987$, and secondary emotion ratings among boys ($M = 1.42$, $SD = 0.45$) were slightly higher than those among girls ($M = 1.35$, $SD = 0.34$), but the difference was not significant, $p = .105$. Consistent with the original mixed-design ANOVA, other main effects and interactions were non-significant.

Table S8. Linear mixed-effects model for trait ratings

Variables	*F*	*p*
**Age Group**	**8.09**	**.005**
Participant Gender	0.55	.459
Age Group × Participant Gender	0.69	.406
Peer Gender	0.51	.475
Peer Gender × Age Group	0.92	.339
Peer Gender × Participant Gender	1.98	.160
Peer Gender × Age Group × Participant Gender	2.35	.126
Peer Gender Conformity	0.67	.414
Peer Gender Conformity × Age Group	0.21	.644
Peer Gender Conformity × Participant Gender	0.01	.934
Peer Gender Conformity × Age Group × Participant Gender	0.51	.476
**Trait Type**	**30.03**	**< .001**
Trait Type × Age Group	1.19	.276
Trait Type × Participant Gender	0.01	.930
Trait Type × Age Group × Participant Gender	3.69	.055
Peer Gender × Peer Gender Conformity	2.81	.094
Peer Gender × Peer Gender Conformity × Age Group	0.60	.438
Peer Gender × Peer Gender Conformity × Participant Gender	0.09	.768
Peer Gender × Peer Gender Conformity × Age Group × Participant Gender	0.84	.360
Peer Gender × Trait Type	0.50	.481
Peer Gender × Trait Type × Age Group	0.19	.663
Peer Gender × Trait Type × Participant Gender	1.05	.306
Peer Gender × Trait Type × Age Group × Participant Gender	0.01	.928
Peer Gender Conformity × Trait Type	0.48	.489
Peer Gender Conformity × Trait Type × Age Group	1.82	.178
Peer Gender Conformity × Trait Type × Participant Gender	2.75	.097
Peer Gender Conformity × Trait Type × Age Group × Participant Gender	0.27	.604
Peer Gender × Peer Gender Conformity × Trait Type	1.66	.198
Peer Gender × Peer Gender Conformity × Trait Type × Age Group	1.52	.217
Peer Gender × Peer Gender Conformity × Trait Type × Participant Gender	0.03	.870
Peer Gender × Peer Gender Conformity × Trait Type × Age Group × Participant Gender	2.95	.086

Consistent with the original mixed-design ANOVA, the main effect of age group was significant, $F(1, 231) = 8.09$, $p = .005$, and the main effect of trait type was significant, $F(1, 1617) = 30.03$, $p < .001$. Other main effects and interactions were non-significant.

Section S4: Psychometric Properties of Measures

Internal Consistency

Table S9. Inter-item correlations and Spearman–Brown reliability coefficients for 2-item emotion subscales, by peer gender, peer gender conformity, emotion type, and emotion valence.

Peer gender	Peer gender conformity	Dependent variable	Inter-item correlation *r*	Spearman–Brown ρ
Boy	GC	Positive primary emotion	.39*	.56
		Negative primary emotion	.42*	.59
		Positive secondary emotion	.38*	.56
		Negative secondary emotion	.41*	.58
Boy	GN	Positive primary emotion	.44*	.61
		Negative primary emotion	.42*	.59
		Positive secondary emotion	.38*	.55
		Negative secondary emotion	.46*	.63
Girl	GC	Positive primary emotion	.35*	.52
		Negative primary emotion	.48*	.65
		Positive secondary emotion	.36*	.53
		Negative secondary emotion	.47*	.64
Girl	GN	Positive primary emotion	.44*	.61
		Negative primary emotion	.52*	.68
		Positive secondary emotion	.34*	.51
		Negative secondary emotion	.54*	.70

Note. GC = gender conforming; GN = gender nonconforming. Each row is based on the two items measuring the specified subscale for the specified target. *$p < .001$.

Table S10. Inter-item correlations and Spearman–Brown reliability coefficients for 2-item trait subscales, by peer gender, peer gender conformity, trait type, and trait valence.

Peer gender	Peer gender conformity	Dependent variable	Inter-item correlation *r*	Spearman–Brown ρ
Boy	GC	Positive UH traits	.43*	.60
		Negative UH traits	.37*	.54
		Positive HN traits	.42*	.59
		Negative HN traits	.40*	.57
Boy	GN	Positive UH traits	.36*	.53
		Negative UH traits	.35*	.52
		Positive HN traits	.36*	.53
		Negative HN traits	.35*	.52
Girl	GC	Positive UH traits	.46*	.63
		Negative UH traits	.41*	.58
		Positive HN traits	.36*	.53
		Negative HN traits	.40*	.57
Girl	GN	Positive UH traits	.40*	.57
		Negative UH traits	.42*	.59
		Positive HN traits	.38*	.55
		Negative HN traits	.44*	.61

Note. GC = gender conforming; GN = gender nonconforming; UH = uniquely human; HN = human nature. Each row is based on the two items measuring the specified subscale for the specified target. *$p < .001$

Convergent and Divergent Validity

Table S11. Within- vs. between-emotion-type mean inter-item correlations, by peer gender, peer gender conformity, and emotion valence.

Peer gender	Peer gender conformity	Dependent variable	Within-emotion-type mean *r*	Between-emotion-type mean *r*
Boy	GC	Positive emotions	.39	.19
		Negative emotions	.41	.27
Boy	GN	Positive emotions	.41	.29
		Negative emotions	.44	.35
Girl	GC	Positive emotions	.35	.22
		Negative emotions	.48	.36
Girl	GN	Positive emotions	.39	.21
		Negative emotions	.53	.39

Note. GC = gender conforming; GN = gender nonconforming. "Within-emotion-type mean *r*" is the mean of Pearson correlations between items belonging to the **same** emotion type at the specified valence (e.g., the mean of the correlation between the two positive primary emotion items and the correlation between the two positive secondary emotion items). "Between-emotion-type mean *r*" is the mean of Pearson correlations between items belonging to **different** emotion types at the specified valence (e.g., the mean of the four pairwise correlations between the two positive primary and the two positive secondary emotion items).

Table S12. Within- vs. between-trait-type mean inter-item correlations, by peer gender, peer gender conformity, and trait valence.

Peer gender	Peer gender conformity	Dependent variable	Within-trait-type mean *r*	Between-trait-type mean *r*
Boy	GC	Positive traits	.42	.28
		Negative traits	.39	.30
Boy	GN	Positive traits	.36	.24
		Negative traits	.35	.29
Girl	GC	Positive traits	.41	.31
		Negative traits	.40	.33
Girl	GN	Positive traits	.39	.33
		Negative traits	.43	.38

Note. GC = gender conforming; GN = gender nonconforming; UH = uniquely human; HN = human nature. "Within-trait-type mean *r*" is the mean of Pearson correlations between items belonging to the **same** trait type at the specified valence (e.g., the mean of the correlation between the two positive UH trait items and the correlation between the two positive HN trait items). "Between-trait-type mean *r*" is the mean of Pearson correlations between items belonging to **different** trait types at the specified valence (e.g., the mean of the four pairwise correlations between the two positive UH trait items and the two positive HN trait items).

Across all 16 comparisons in Tables S11 and S12, within-type mean correlations consistently exceeded between-type mean correlations. This pattern provides evidence that participants' ratings are consistent with the emotion type (primary vs. secondary) and trait type (UH vs. HN) structure of the measures and provides descriptive convergent and discriminant validity evidence that humanness constitutes a coherent judgment dimension over and above shared valence.

Section S5: Analyses Including Valence as a Within-Subjects Factor

Emotion Valence

A six-way mixed-design ANOVA was conducted for emotion ratings. In this analysis, participant gender (boy vs. girl) and age group (9–10 years vs. 11–12 years) were included as between-subjects variables, while peer gender (boy vs. girl), peer gender conformity (GC vs. GN), emotion type (primary vs. secondary), and emotion valence (positive vs. negative) were included as within-subjects variables.

Table S13. ANOVA results for emotion ratings, including emotion valence as a within-subjects factor

Variables	*F*	*p*	η_p^2
**Age Group**	**10.26**	**.002**	**.04**
Participant Gender	0.77	.380	.00
**Age Group × Participant Gender**	**5.77**	**.017**	**.02**
Peer Gender	3.82	.052	.02
Peer Gender × Age Group	3.15	.077	.01
Peer Gender × Participant Gender	1.85	.175	.01
Peer Gender × Age Group × Participant Gender	3.56	.061	.02
Peer Gender Conformity	0.00	.990	.00
Peer Gender Conformity × Age Group	0.00	.948	.00
Peer Gender Conformity × Participant Gender	2.36	.126	.01
Peer Gender Conformity × Age Group × Participant Gender	0.02	.887	.00
**Emotion Type**	**191.15**	**< .001**	**.45**
Emotion Type × Age Group	0.96	.329	.00
Emotion Type × Participant Gender	2.20	.140	.01
Emotion Type × Age Group × Participant Gender	0.12	.731	.00
**Emotion Valence**	**332.99**	**< .001**	**.59**
Emotion Valence × Age Group	0.29	.591	.00
Emotion Valence × Participant Gender	1.88	.171	.01
Emotion Valence × Age Group × Participant Gender	0.13	.722	.00
**Peer Gender × Peer Gender Conformity**	**7.56**	**.006**	**.03**
Peer Gender × Peer Gender Conformity × Age Group	0.36	.547	.00
Peer Gender × Peer Gender Conformity × Participant Gender	1.43	.233	.01
Peer Gender × Peer Gender Conformity × Age Group × Participant Gender	0.07	.789	.00
Peer Gender × Emotion Type	0.01	.932	.00
Peer Gender × Emotion Type × Age Group	0.12	.727	.00
Peer Gender × Emotion Type × Participant Gender	0.01	.932	.00
Peer Gender × Emotion Type × Age Group × Participant Gender	0.02	.880	.00
Peer Gender Conformity × Emotion Type	0.00	.952	.00
Peer Gender Conformity × Emotion Type × Age Group	0.02	.893	.00
Peer Gender Conformity × Emotion Type × Participant Gender	0.06	.810	.00
Peer Gender Conformity × Emotion Type × Age Group × Participant Gender	0.66	.418	.00
**Peer Gender × Peer Gender Conformity × Emotion Type**	**14.45**	**< .001**	**.06**
Peer Gender × Peer Gender Conformity × Emotion Type × Age Group	0.01	.929	.00
Peer Gender × Peer Gender Conformity × Emotion Type × Participant Gender	1.03	.311	.00
Peer Gender × Peer Gender Conformity × Emotion Type × Age Group × Participant Gender	3.33	.069	.01
Peer Gender × Emotion Valence	3.58	.060	.02
Peer Gender × Emotion Valence × Age Group	0.06	.810	.00
Peer Gender × Emotion Valence × Participant Gender	0.81	.369	.00
Peer Gender × Emotion Valence × Age Group × Participant Gender	0.42	.519	.00
**Peer Gender Conformity × Emotion Valence**	**99.65**	**< .001**	**.30**
**Peer Gender Conformity × Emotion Valence × Age Group**	**18.71**	**< .001**	**.08**
Peer Gender Conformity × Emotion Valence × Participant Gender	0.00	.999	.00
Peer Gender Conformity × Emotion Valence × Age Group × Participant Gender	0.00	.999	.00
**Peer Gender × Peer Gender Conformity × Emotion Valence**	**34.76**	**< .001**	**.13**

Peer Gender × Peer Gender Conformity × Emotion Valence × Age Group	0.43	.513	.00
Peer Gender × Peer Gender Conformity × Emotion Valence × Participant Gender	0.94	.333	.00
Peer Gender × Peer Gender Conformity × Emotion Valence × Age Group × Participant Gender	0.03	.859	.00
Emotion Type × Emotion Valence	3.68	.056	.02
Emotion Type × Emotion Valence × Age Group	3.68	.056	.02
Emotion Type × Emotion Valence × Participant Gender	0.27	.606	.00
Emotion Type × Emotion Valence × Age Group × Participant Gender	0.46	.498	.00
Peer Gender × Emotion Type × Emotion Valence	2.07	.152	.01
Peer Gender × Emotion Type × Emotion Valence × Age Group	0.25	.621	.00
Peer Gender × Emotion Type × Emotion Valence × Participant Gender	0.47	.496	.00
Peer Gender × Emotion Type × Emotion Valence × Age Group × Participant Gender	0.02	.895	.00
Peer Gender Conformity × Emotion Type × Emotion Valence	1.84	.176	.01
Peer Gender Conformity × Emotion Type × Emotion Valence × Age Group	0.56	.457	.00
Peer Gender Conformity × Emotion Type × Emotion Valence × Participant Gender	0.16	.689	.00
Peer Gender Conformity × Emotion Type × Emotion Valence × Age Group × Participant Gender	0.13	.717	.00
Peer Gender × Peer Gender Conformity × Emotion Type × Emotion Valence	0.00	.957	.00
Peer Gender × Peer Gender Conformity × Emotion Type × Emotion Valence × Age Group	0.55	.458	.00
Peer Gender × Peer Gender Conformity × Emotion Type × Emotion Valence × Participant Gender	1.30	.255	.01
Peer Gender × Peer Gender Conformity × Emotion Type × Emotion Valence × Age Group × Participant Gender	0.21	.651	.00

When emotion valence was included in the analyses, the main effect of emotion valence was significant, $F(1, 231) = 332.99$, $p < .001$, $\eta_p^2 = .59$. Ratings of positive emotions ($M = 1.86$, $SD = 0.37$) were significantly higher than those of negative emotions ($M = 1.23$, $SD = 0.43$). The peer gender conformity × emotion valence interaction was also significant, $F(1, 231) = 99.65$, $p < .001$, $\eta_p^2 = .30$. Post hoc analyses were conducted separately by emotion valence. Ratings of positive emotions were significantly higher for GC peers ($M = 2.02$, $SD = 0.43$) than for GN peers ($M = 1.71$, $SD = 0.47$), $p < .001$, $d = 0.69$; ratings of negative emotions were significantly higher for GN peers ($M = 1.38$, $SD = 0.55$) than for GC peers ($M = 1.07$, $SD = 0.48$), $p < .001$, $d = 0.60$. These effects were qualified in a significant peer gender conformity × emotion valence × peer gender interaction, $F(1, 231) = 34.76$, $p < .001$, $\eta_p^2 = .13$, and in a significant peer gender conformity × emotion valence × age group interaction, $F(1, 231) = 18.71$, $p < .001$, $\eta_p^2 = .08$.

To examine the significant peer gender conformity × emotion valence × peer gender interaction, post hoc simple interaction analyses were conducted separately for each peer gender condition. For peer girls, the peer gender conformity × emotion valence interaction was significant, $F(1, 231) = 21.98$, $p < .001$, $\eta_p^2 = .09$. Ratings of positive emotions were significantly higher for GC girls ($M = 2.01$, $SD = 0.49$) than for GN girls ($M = 1.79$, $SD = 0.54$), $p < .001$, $d = 0.45$. Ratings of negative emotions were significantly higher for GN girls ($M = 1.29$, $SD = 0.64$) than for GC girls ($M = 1.16$, $SD = 0.59$), $p = .009$, $d = 0.21$. For peer boys, the peer gender conformity × emotion valence interaction was substantially larger, $F(1, 231) = 132.53$, $p < .001$, $\eta_p^2 = .37$. Ratings of positive emotions were significantly higher for GC boys ($M = 2.02$, $SD = 0.50$) than for GN boys ($M = 1.64$, $SD = 0.57$), $p < .001$, $d = 0.72$, whereas ratings of negative emotions were significantly higher for GN boys ($M = 1.48$, $SD = 0.64$) than for GC boys ($M = 0.99$, $SD = 0.54$), $p < .001$, $d = 0.82$. This indicates that school-aged children perceive GN peers as experiencing fewer positive and more negative emotions than GC peers, and this pattern is stronger for peer boys than for peer girls.

To examine the significant peer gender conformity × emotion valence × age group interaction, post hoc simple interaction analyses were conducted separately for each age group. Among participants in the 9–10 years age group, the peer gender conformity × emotion valence interaction was significant, $F(1, 231) = 15.94$, $p < .001$, $\eta_p^2 = .07$. Ratings of positive emotions were significantly higher for GC peers ($M = 1.90$, $SD = 0.42$) than for

GN peers (M = 1.73, SD = 0.45), p < .001, d = 0.40. Conversely, ratings of negative emotions were significantly higher for GN peers (M = 1.24, SD = 0.48) than for GC peers (M = 1.07, SD = 0.45), p < .001, d = 0.38. Among participants in the 11–12 years age group, the peer gender conformity × emotion valence interaction was stronger, $F(1, 231) = 102.79$, p < .001, $\eta_p^2 = .31$. Ratings of positive emotions were significantly higher for GC peers (M = 2.14, SD = 0.40) than for GN peers (M = 1.69, SD = 0.49), p < .001, d = 0.98, whereas ratings of negative emotions were significantly higher for GN peers (M = 1.52, SD = 0.57) than for GC peers (M = 1.08, SD = 0.51), p < .001, d = 0.81. This indicates that school-aged children, especially older children, perceive GN peers as experiencing fewer positive and more negative emotions than GC peers.

It is noteworthy that the peer gender conformity × emotion valence × emotion type interaction was not significant, $F(1, 231) = 1.84$, p = .176, $\eta_p^2 = .01$. The peer gender conformity × emotion valence interaction was significant for both primary emotions, $F(1, 231) = 81.49$, p < .001, $\eta_p^2 = .26$, and secondary emotions, $F(1, 231) = 73.01$, p < .001, $\eta_p^2 = .24$. Positive emotion ratings for GC peers were significantly higher than those for GN peers, including both positive primary emotions (GC: M = 2.21, SD = 0.50; GN: M = 1.88, SD = 0.57), p < .001, d = 0.62, and positive secondary emotions (GC: M = 1.83, SD = 0.53; GN: M = 1.54, SD = 0.54), p < .001, d = 0.54. Negative emotion ratings for GN peers were significantly higher than those for GC peers, including both negative primary emotions (GC: M = 1.20, SD = 0.51; GN: M = 1.54, SD = 0.55), p < .001, d = 0.63, and negative secondary emotions (GC: M = 0.94, SD = 0.59; GN: M = 1.23, SD = 0.69), p < .001, d = 0.44. Consistent with the primary analyses reported in the main text, these supplemental findings do not support the infrahumanization perspective. In the present study, children did not attribute fewer secondary emotions, both positive and negative, to GN peers than to GC peers; instead, children attributed fewer positive emotions and more negative emotions to GN peers than to GC peers, regardless of emotion type/humanness.

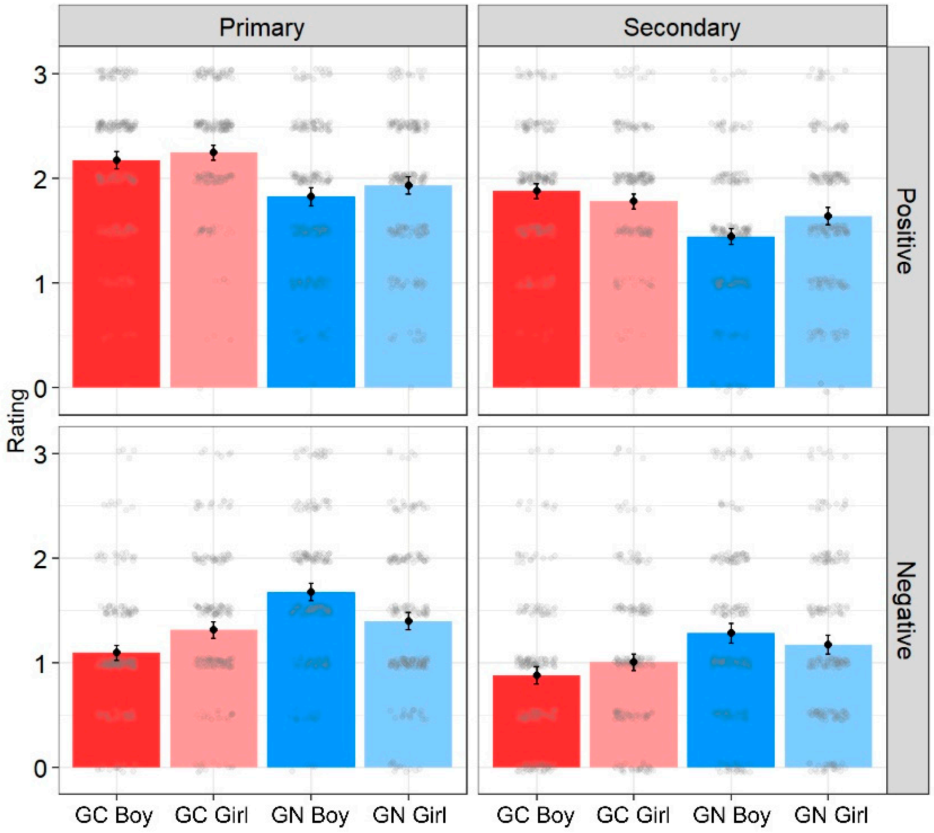

Figure S1. Mean emotion ratings as a function of peer gender, peer gender conformity, emotion type, and emotion valence. GC = gender conforming; GN = gender nonconforming. Error bars represent 95% confidence intervals.

Trait Valence

A six-way mixed-design ANOVA was conducted for trait ratings. In this analysis, participant gender (boy vs. girl) and age group (9–10 years vs. 11–12 years) were included as between-subjects variables, while peer gender (boy vs. girl), peer gender conformity (GC vs. GN), trait type (UH vs. HN), and trait valence (positive vs. negative) were included as within-subjects variables.

Table S14. ANOVA results for trait ratings, including trait valence as a within-subjects factor

Variables	F	p	η_p^2
**Age Group**	**8.09**	**.005**	**.03**
Participant Gender	0.55	.459	.00
Age Group × Participant Gender	0.69	.406	.00
Peer Gender	0.48	.490	.00
Peer Gender × Age Group	0.86	.356	.00
Peer Gender × Participant Gender	1.85	.175	.01
Peer Gender × Age Group × Participant Gender	2.19	.140	.01
Peer Gender Conformity	0.78	.377	.00
Peer Gender Conformity × Age Group	0.25	.617	.00
Peer Gender Conformity × Participant Gender	0.01	.929	.00
Peer Gender Conformity × Age Group × Participant Gender	0.60	.440	.00
**Trait Type**	**24.14**	**< .001**	**.10**
Trait Type × Age Group	0.96	.329	.00
Trait Type × Participant Gender	0.01	.937	.00
Trait Type × Age Group × Participant Gender	2.96	.087	.01
**Trait Valence**	**213.41**	**< .001**	**.48**
Trait Valence × Age Group	0.02	.878	.00
Trait Valence × Participant Gender	1.85	.175	.01
Trait Valence × Age Group × Participant Gender	3.30	.071	.01
Peer Gender × Peer Gender Conformity	2.62	.107	.01
Peer Gender × Peer Gender Conformity × Age Group	0.56	.455	.00
Peer Gender × Peer Gender Conformity × Participant Gender	0.08	.776	.00
Peer Gender × Peer Gender Conformity × Age Group × Participant Gender	0.78	.379	.00
Peer Gender × Trait Type	0.52	.472	.00
Peer Gender × Trait Type × Age Group	0.20	.656	.00
Peer Gender × Trait Type × Participant Gender	1.10	.296	.01
Peer Gender × Trait Type × Age Group × Participant Gender	0.01	.927	.00
Peer Gender Conformity × Trait Type	0.61	.437	.00
Peer Gender Conformity × Trait Type × Age Group	2.30	.131	.01
Peer Gender Conformity × Trait Type × Participant Gender	3.48	.063	.02
Peer Gender Conformity × Trait Type × Age Group × Participant Gender	0.34	.560	.00
Peer Gender × Peer Gender Conformity × Trait Type	1.64	.202	.01
Peer Gender × Peer Gender Conformity × Trait Type × Age Group	1.50	.222	.01
Peer Gender × Peer Gender Conformity × Trait Type × Participant Gender	0.03	.871	.00
Peer Gender × Peer Gender Conformity × Trait Type × Age Group × Participant Gender	2.91	.089	.01
**Peer Gender × Trait Valence**	**19.76**	**< .001**	**.08**
Peer Gender × Trait Valence × Age Group	0.00	.983	.00
Peer Gender × Trait Valence × Participant Gender	1.81	.180	.01
Peer Gender × Trait Valence × Age Group × Participant Gender	0.06	.806	.00
Peer Gender Conformity × Trait Valence	0.03	.870	.00
Peer Gender Conformity × Trait Valence × Age Group	0.56	.456	.00
Peer Gender Conformity × Trait Valence × Participant Gender	3.82	.052	.02
Peer Gender Conformity × Trait Valence × Age Group × Participant Gender	1.37	.243	.01
**Peer Gender × Peer Gender Conformity × Trait Valence**	**138.12**	**< .001**	**.37**
Peer Gender × Peer Gender Conformity × Trait Valence × Age Group	0.13	.717	.00
Peer Gender × Peer Gender Conformity × Trait Valence × Participant Gender	1.74	.189	.01
Peer Gender × Peer Gender Conformity × Trait Valence × Age Group × Participant Gender	0.61	.436	.00

Trait Type × Trait Valence	1.29	.257	.01
Trait Type × Trait Valence × Age Group	0.06	.808	.00
Trait Type × Trait Valence × Participant Gender	0.09	.761	.00
Trait Type × Trait Valence × Age Group × Participant Gender	2.04	.155	.01
Peer Gender × Trait Type × Trait Valence	0.04	.850	.00
Peer Gender × Trait Type × Trait Valence × Age Group	1.07	.302	.01
Peer Gender × Trait Type × Trait Valence × Participant Gender	0.05	.829	.00
Peer Gender × Trait Type × Trait Valence × Age Group × Participant Gender	0.03	.856	.00
Peer Gender Conformity × Trait Type × Trait Valence	1.47	.226	.01
Peer Gender Conformity × Trait Type × Trait Valence × Age Group	0.96	.328	.00
Peer Gender Conformity × Trait Type × Trait Valence × Participant Gender	0.30	.587	.00
Peer Gender Conformity × Trait Type × Trait Valence × Age Group × Participant Gender	0.19	.666	.00
Peer Gender × Peer Gender Conformity × Trait Type × Trait Valence	0.01	.933	.00
Peer Gender × Peer Gender Conformity × Trait Type × Trait Valence × Age Group	0.17	.683	.00
Peer Gender × Peer Gender Conformity × Trait Type × Trait Valence × Participant Gender	0.33	.568	.00
Peer Gender × Peer Gender Conformity × Trait Type × Trait Valence × Age Group × Participant Gender	1.68	.196	.01

The main effect of trait valence was significant, $F(1, 231) = 213.41$, $p < .001$, $\eta^2_p = .48$. Ratings of positive traits ($M = 1.90$, $SD = 0.35$) were significantly higher than those of negative traits ($M = 1.34$, $SD = 0.39$). The interaction between peer gender and trait valence was significant, $F(1, 231) = 19.76$, $p < .001$, $\eta^2_p = .08$. Specifically, girl peers ($M = 1.95$, $SD = 0.41$) were attributed significantly more positive traits than boy peers ($M = 1.85$, $SD = 0.40$), $p < .001$, $d = 0.23$, and girl peers ($M = 1.29$, $SD = 0.45$) were attributed significantly fewer negative traits than boy peers ($M = 1.40$, $SD = 0.45$), $p < .001$, $d = -0.26$.

The lower-order effects above were qualified in a significant peer gender × peer gender conformity × trait valence interaction, $F(1, 231) = 138.12$, $p < .001$, $\eta^2_p = .37$. Post hoc analyses were conducted separately by peer gender. For peer boys, the peer gender conformity × trait valence interaction was significant, $F(1, 231) = 86.32$, $p < .001$, $\eta^2_p = .27$. Ratings of positive traits were significantly higher for GN boys ($M = 2.03$, $SD = 0.50$) than for GC boys ($M = 1.68$, $SD = 0.55$), $p < .001$, $d = 0.65$, whereas ratings of negative traits were significantly higher for GC boys ($M = 1.59$, $SD = 0.57$) than for GN boys ($M = 1.22$, $SD = 0.54$), $p < .001$, $d = 0.66$. For peer girls, the peer gender conformity × trait valence interaction was also significant, $F(1, 231) = 79.63$, $p < .001$, $\eta^2_p = .26$, but the interaction pattern was in the opposite direction. Ratings of positive traits were significantly higher for GC girls ($M = 2.11$, $SD = 0.51$) than for GN girls ($M = 1.79$, $SD = 0.55$), $p < .001$, $d = 0.61$, whereas ratings of negative traits were significantly higher for GN girls ($M = 1.49$, $SD = 0.61$) than for GC girls ($M = 1.08$, $SD = 0.56$), $p < .001$, $d = 0.69$. Consistent with the primary analyses reported in the main text, these supplemental findings do not support the trait-based dehumanization perspective. In the present study, on the whole, across peer genders, children did not attribute fewer UH/HN traits to GN peers than to GC peers.

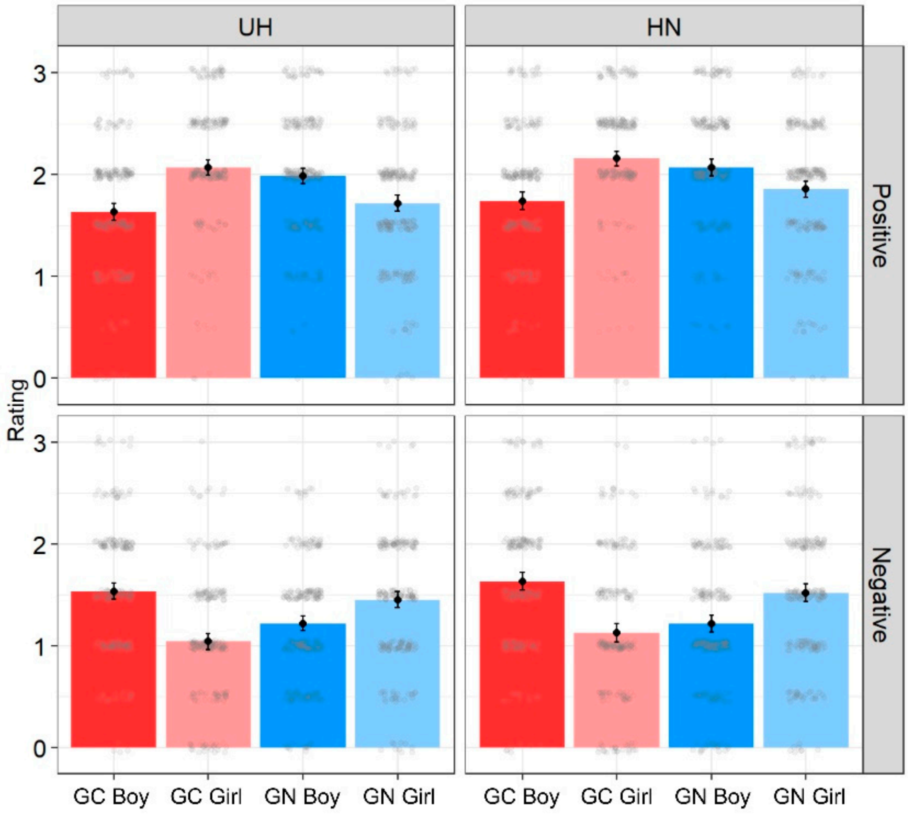

Figure S2. Mean trait ratings as a function of peer gender, peer gender conformity, trait type, and trait valence. UH = uniquely human; HN = human nature; GC = gender conforming; GN = gender nonconforming. Error bars represent 95% confidence intervals.

Section S6: Bayesian Analyses for Focal GC–GN Comparisons

Following a suggestion by an anonymous reviewer, Bayesian paired-sample *t*-tests were conducted to supplement the frequentist analyses for the contrasts most central to the infrahumanization and trait-based dehumanization hypotheses: comparisons of GC and GN peer ratings on secondary emotions and on human traits (human traits combining UH and HN traits, UH traits, and HN traits). Whereas frequentist null-hypothesis significance tests (e.g., ANOVA) can only reject or fail to reject the null hypothesis, Bayesian analyses directly compare the likelihood of the observed data under competing hypotheses, thereby allowing evaluation of whether a non-significant frequentist result reflects support for the null hypothesis or merely insufficient evidence to distinguish between null and alternative hypotheses (Wagenmakers et al., 2018). Specifically, two competing a priori hypotheses were contrasted: the directional infrahumanization or trait-based dehumanization hypothesis (H_-: $\delta < 0$), which predicts lower ratings for GN than for GC peers, and the null hypothesis (H_0: $\delta = 0$), which predicts equivalent ratings between GC and GN peers.

Bayes factors are reported as BF_{0-}, indexing the relative evidence for H_0 over H_-; values greater than 1 favor H_0, values less than 1 favor H_-, and $BF_{-0} = 1/BF_{0-}$. Following established guidelines (Jeffreys, 1998; Wetzels et al., 2011), BF_{0-} values of 1–3 = “anecdotal evidence”, 3–10 = “moderate evidence”, 10–30 = “strong evidence”, 30–100 = “very strong evidence”, and >100 = “extreme evidence” in favor of H_0 over H_-. Specifically, a BF_{0-} value of 3 or above indicates varying degrees of support for the null hypothesis. All Bayesian analyses were conducted in JASP version 0.98.1 (JASP Team, 2026), using the default Jeffreys–Zellner–Siow prior specification (Rouder et al., 2009). Specifically, the analyses employed a point null (H_0: $\delta = 0$) and a half-Cauchy prior on the standardized effect size (scale $r = 0.707$, JASP’s default medium width) truncated to negative values to reflect the directional alternative hypotheses (H_-: $\delta < 0$).

Emotion Ratings

For the contrast between secondary emotion ratings for GC peers and GN peers, $BF_{0-} = 13.11$ (posterior median $\delta = –0.045$, 95% CrI [–0.147, –0.002]), indicating that the observed data were 13.11 times more likely to align with the null hypothesis than with the infrahumanization hypothesis. This provides strong evidence that children attributed secondary emotions comparably to GC and GN peers. Robustness checks confirmed that these conclusions were stable across wider prior specifications (see below). Together with the non-significant results in frequentist tests, these results provide converging evidence that Chinese children in this sample did not attribute fewer secondary emotions to GN peers than to GC peers, consistent with the null hypothesis.

Figure S3. Robustness checks for Bayesian analyses of GC–GN contrasts in secondary emotions ratings

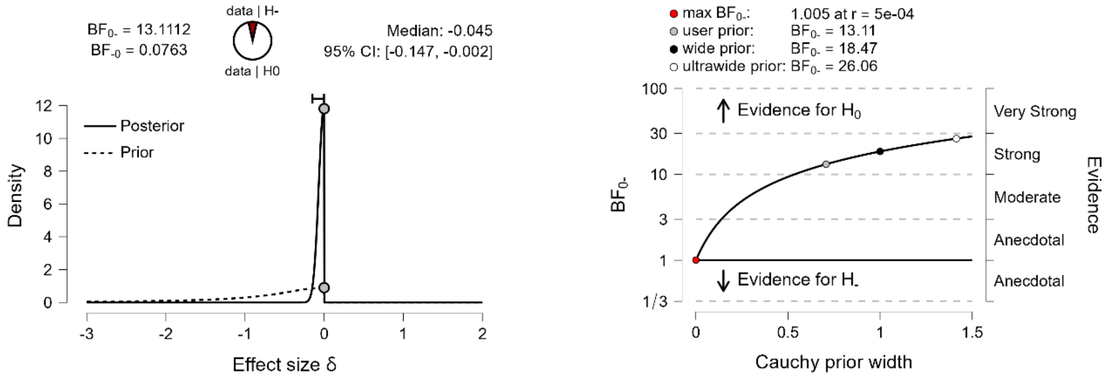

Trait ratings

For the contrast between human trait ratings for GC peers and GN peers, $BF_{0-} = 24.55$ (posterior median $\delta = –0.028$, 95% CrI [–0.111, –0.001]), indicating that the observed data were 24.55 times more likely to align with the null hypothesis than with the trait-based dehumanization hypothesis. This provides strong evidence that children attributed human traits comparably to GC and GN peers. Bayesian tests were also conducted separately for each type of human traits. For UH traits, $BF_{0-} = 29.12$ (posterior median $\delta = –0.024$, 95% CrI [–0.101, –0.001]), indicating strong evidence for H_0; for HN traits, $BF_{0-} = 14.84$ (posterior median $\delta = –0.041$, 95% CrI [–

0.140, –0.002]), also indicating strong evidence for H_0. Robustness checks confirmed that these conclusions were stable across wider prior specifications (see below). Together with the non-significant results in frequentist tests, these results provide converging evidence that Chinese children in this sample did not attribute fewer human traits (neither UH traits nor HN traits) to GN peers than to GC peers, consistent with the null hypothesis.

Figure S4. Robustness checks for Bayesian analyses of GC–GN contrasts in (a) human trait aggregate, (b) UH trait, and (c) HN trait ratings

(a)

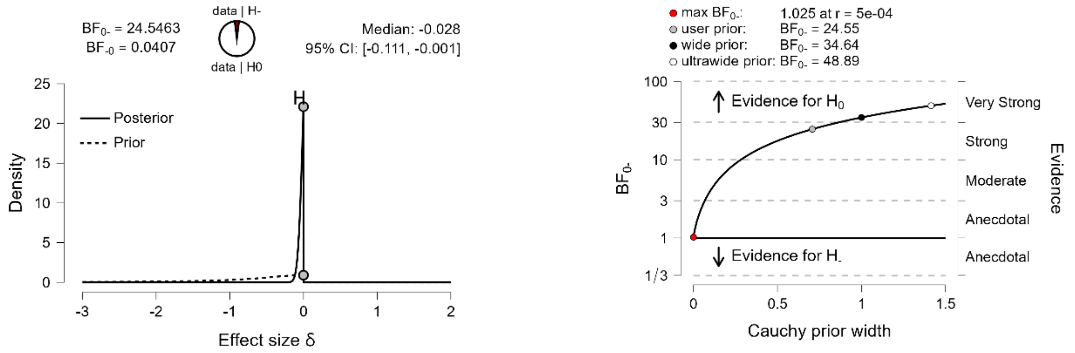

(b)

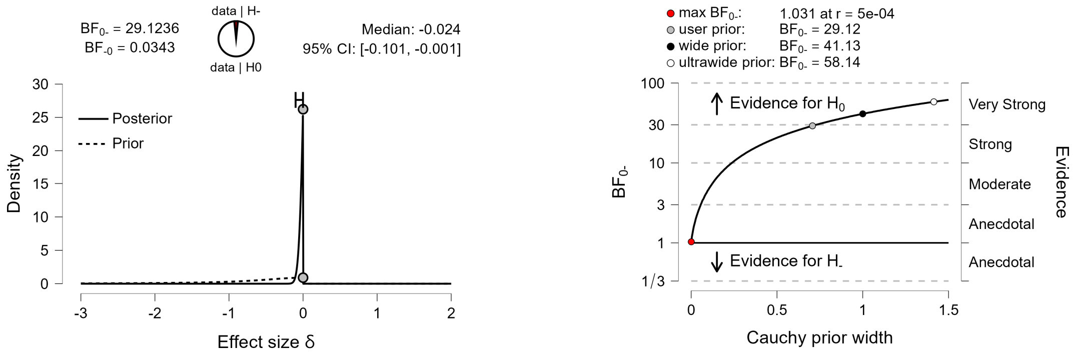

(c)

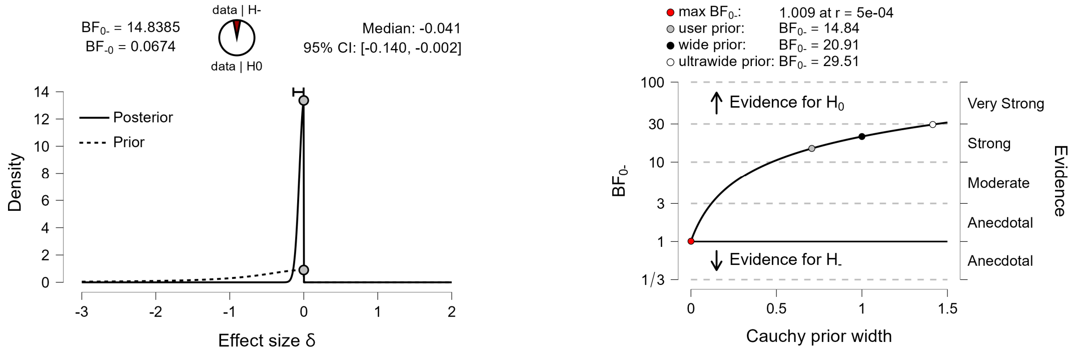

Section S7: Sensitivity Analyses for Stimulus Comprehension

During the rating tasks, experimenters recorded whether each participant requested clarification of any emotion or trait word. Standardized definitions were provided upon request. Across the full sample, 45 of 235 children (19.1%) requested clarification for at least one emotion word, and 28 of 235 (11.9%) requested clarification for at least one trait word. Clarification requests were more common in the 9–10y age group (emotion: 31/117, 26.5%; trait: 25/117, 21.4%) than in the 11–12y age group (emotion: 14/118, 11.9%; trait: 3/118, 2.5%); chi-square tests revealed that both differences were statistically significant, $\chi^2(1, N = 235) = 8.12$, $p = .004$ for emotion words; $\chi^2(1, N = 235) = 19.84$, $p < .001$ for trait words.

To examine whether the null findings in the present study were potentially driven by children who did not understand the emotion/trait stimuli, supplementary ANOVAs excluding children who requested clarification for any emotion or trait word were conducted ($N = 45$ excluded for the ANOVA on emotion ratings; $N = 28$ excluded for the ANOVA on trait ratings).

For emotion ratings, consistent with the original analyses, the supplementary analyses revealed a significant peer gender × peer gender conformity × emotion type interaction, $F(1, 186) = 13.38$, $p < .001$, $\eta_p^2 = .07$. Post-hoc analyses similarly revealed a significant peer gender × peer gender conformity interaction for primary emotions, $F(1, 186) = 20.30$, $p < .001$, $\eta_p^2 = .10$, such that primary emotion ratings for GN boys ($M = 1.76$, $SD = 0.41$) were significantly greater than those for GC boys ($M = 1.65$, $SD = 0.42$), $p = .003$, and primary emotion ratings for GC girls ($M = 1.80$, $SD = 0.43$) were significantly greater than those for GN girls ($M = 1.66$, $SD = 0.39$), $p < .001$, and a non-significant interaction for secondary emotions, $F(1, 186) = 0.47$, $p = .495$, $\eta_p^2 = .00$, where secondary emotion ratings for GC boys ($M = 1.40$, $SD = 0.50$) and GN boys ($M = 1.37$, $SD = 0.55$) did not significantly differ, $p = .403$, and those for GC girls ($M = 1.41$, $SD = 0.49$) and GN girls ($M = 1.42$, $SD = 0.54$) did not significantly differ, $p = .817$.

For trait ratings, consistent with the original analyses, the supplementary analyses revealed a non-significant peer gender conformity main effect, $F(1, 203) = 0.79$, $p = .375$, $\eta_p^2 = .00$. Overall human trait ratings for GC peers ($M = 1.63$, $SD = 0.24$) did not significantly differ from those for GN peers ($M = 1.64$, $SD = 0.24$). The peer gender conformity × trait type interaction was also non-significant, $F(1, 203) = 0.85$, $p = .358$, $\eta_p^2 = .00$. UH trait ratings for GC peers ($M = 1.58$, $SD = 0.29$) did not significantly differ from those for GN peers ($M = 1.61$, $SD = 0.30$), $p = .185$; HN trait ratings for GC peers ($M = 1.67$, $SD = 0.30$) did not significantly differ from those for GN peers ($M = 1.67$, $SD = 0.28$), $p = .995$.

These sensitivity analyses suggest that the null dehumanization findings reported in the main text are unlikely to be driven by participants who did not understand emotion or trait stimuli. In addition, standardized definitions were provided to participants who indicated they did not understand some of the stimuli, mitigating the impact of comprehension difficulties on the results.

Section S8: Analyses Including Task Order as a Between-Subjects Factor

A six-way mixed-design ANOVA was conducted for emotion and trait ratings, respectively. In these analyses, participant gender (boy vs. girl), age group (9–10 years vs. 11–12 years), and task order (0 = emotion attribution task first, 1 = trait attribution task first) were included as between-subjects variables, while peer gender (boy vs. girl), peer gender conformity (GC vs. GN), and emotion/trait type (primary vs. secondary; UH vs. HN) were included as within-subjects variables.

Table S15. ANOVA results for emotion ratings, including task order as a between-subjects factor

**Variables**	***F***	***p***	η_p^2
Participant Gender	0.83	.364	.00
**Age Group**	**9.93**	**.002**	**.04**
Task Order	0.77	.381	.00
**Age Group × Participant Gender**	**6.14**	**.014**	**.03**
Task Order × Participant Gender	1.01	.316	.00
Age Group × Task Order	0.18	.668	.00
Age Group × Task Order × Participant Gender	0.31	.577	.00
**Peer Gender**	**4.17**	**.042**	**.02**
Peer Gender × Participant Gender	1.67	.197	.01
Peer Gender × Age Group	2.98	.086	.01
Peer Gender × Task Order	3.04	.083	.01
Peer Gender × Age Group × Participant Gender	3.67	.057	.02
Peer Gender × Task Order × Participant Gender	0.51	.478	.00
Peer Gender × Age Group × Task Order	1.08	.300	.01
Peer Gender × Age Group × Task Order × Participant Gender	0.00	.951	.00
Peer Gender Conformity	0.01	.907	.00
Peer Gender Conformity × Participant Gender	2.65	.105	.01
Peer Gender Conformity × Age Group	0.02	.895	.00
Peer Gender Conformity × Task Order	1.61	.206	.01
Peer Gender Conformity × Age Group × Participant Gender	0.00	.989	.00
Peer Gender Conformity × Task Order × Participant Gender	1.51	.220	.01
Peer Gender Conformity × Age Group × Task Order	1.63	.203	.01
Peer Gender Conformity × Age Group × Task Order × Participant Gender	0.05	.823	.00
**Emotion Type**	**191.06**	**< .001**	**.46**
Emotion Type × Participant Gender	2.24	.136	.01
Emotion Type × Age Group	1.01	.316	.00
Emotion Type × Task Order	0.33	.569	.00
Emotion Type × Age Group × Participant Gender	0.05	.820	.00
Emotion Type × Task Order × Participant Gender	0.39	.533	.00
Emotion Type × Age Group × Task Order	1.55	.215	.01
Emotion Type × Age Group × Task Order × Participant Gender	1.38	.241	.01
**Peer Gender × Peer Gender Conformity**	**6.93**	**.009**	**.03**
Peer Gender × Peer Gender Conformity × Participant Gender	1.51	.220	.01
Peer Gender × Peer Gender Conformity × Age Group	0.35	.554	.00
Peer Gender × Peer Gender Conformity × Task Order	0.27	.606	.00
Peer Gender × Peer Gender Conformity × Age Group × Participant Gender	0.14	.711	.00
Peer Gender × Peer Gender Conformity × Task Order × Participant Gender	2.95	.087	.01
Peer Gender × Peer Gender Conformity × Age Group × Task Order	0.80	.371	.00
Peer Gender × Peer Gender Conformity × Age Group × Task Order × Participant Gender	0.42	.519	.00
Peer Gender × Emotion Type	0.03	.869	.00
Peer Gender × Emotion Type × Participant Gender	0.00	.971	.00
Peer Gender × Emotion Type × Age Group	0.12	.734	.00
Peer Gender × Emotion Type × Task Order	0.02	.896	.00
Peer Gender × Emotion Type × Age Group × Participant Gender	0.06	.810	.00
Peer Gender × Emotion Type × Task Order × Participant Gender	0.04	.845	.00

Peer Gender × Emotion Type × Age Group × Task Order	2.51	.114	.01
Peer Gender × Emotion Type × Age Group × Task Order × Participant Gender	0.07	.798	.00
Peer Gender Conformity × Emotion Type	0.00	.983	.00
Peer Gender Conformity × Emotion Type × Participant Gender	0.04	.834	.00
Peer Gender Conformity × Emotion Type × Age Group	0.03	.859	.00
Peer Gender Conformity × Emotion Type × Task Order	0.04	.833	.00
Peer Gender Conformity × Emotion Type × Age Group × Participant Gender	0.53	.466	.00
Peer Gender Conformity × Emotion Type × Task Order × Participant Gender	1.02	.314	.00
Peer Gender Conformity × Emotion Type × Age Group × Task Order	0.29	.592	.00
Peer Gender Conformity × Emotion Type × Age Group × Task Order × Participant Gender	0.03	.866	.00
**Peer Gender × Peer Gender Conformity × Emotion Type**	**14.46**	**< .001**	**.06**
Peer Gender × Peer Gender Conformity × Emotion Type × Participant Gender	0.97	.326	.00
Peer Gender × Peer Gender Conformity × Emotion Type × Age Group	0.01	.943	.00
Peer Gender × Peer Gender Conformity × Emotion Type × Task Order	0.04	.840	.00
Peer Gender × Peer Gender Conformity × Emotion Type × Age Group × Participant Gender	3.42	.066	.02
Peer Gender × Peer Gender Conformity × Emotion Type × Task Order × Participant Gender	0.41	.525	.00
Peer Gender × Peer Gender Conformity × Emotion Type × Age Group × Task Order	0.15	.697	.00
Peer Gender × Peer Gender Conformity × Emotion Type × Age Group × Task Order × Participant Gender	0.21	.648	.00

Table S16. ANOVA results for trait ratings, including task order as a between-subjects factor

**Variables**	F	p	η_p^2
Participant Gender	0.57	.453	.00
**Age Group**	**7.93**	**.005**	**.03**
Task Order	1.37	.242	.01
Age Group × Participant Gender	0.64	.426	.00
Task Order × Participant Gender	0.01	.906	.00
Age Group × Task Order	0.06	.815	.00
Age Group × Task Order × Participant Gender	1.25	.264	.01
Peer Gender	0.50	.482	.00
Peer Gender × Participant Gender	1.67	.197	.01
Peer Gender × Age Group	0.70	.405	.00
Peer Gender × Task Order	0.06	.810	.00
Peer Gender × Age Group × Participant Gender	2.24	.135	.01
Peer Gender × Task Order × Participant Gender	0.03	.871	.00
Peer Gender × Age Group × Task Order	0.15	.703	.00
Peer Gender × Age Group × Task Order × Participant Gender	2.01	.157	.01
Peer Gender Conformity	0.75	.389	.00
Peer Gender Conformity × Participant Gender	0.01	.927	.00
Peer Gender Conformity × Age Group	0.19	.664	.00
Peer Gender Conformity × Task Order	0.13	.724	.00
Peer Gender Conformity × Age Group × Participant Gender	0.68	.411	.00
Peer Gender Conformity × Task Order × Participant Gender	0.09	.769	.00
Peer Gender Conformity × Age Group × Task Order	0.85	.357	.00
Peer Gender Conformity × Age Group × Task Order × Participant Gender	1.36	.244	.01
**Trait Type**	**25.31**	**< .001**	**.10**
Trait Type × Participant Gender	0.01	.929	.00
Trait Type × Age Group	0.87	.352	.00
Trait Type × Task Order	1.86	.174	.01
Trait Type × Age Group × Participant Gender	3.05	.082	.01
Trait Type × Task Order × Participant Gender	0.86	.355	.00
Trait Type × Age Group × Task Order	0.47	.495	.00
Trait Type × Age Group × Task Order × Participant Gender	1.70	.194	.01
Peer Gender × Peer Gender Conformity	2.90	.090	.01
Peer Gender × Peer Gender Conformity × Participant Gender	0.05	.827	.00

Peer Gender × Peer Gender Conformity × Age Group	0.70	.402	.00
Peer Gender × Peer Gender Conformity × Task Order	1.36	.244	.01
Peer Gender × Peer Gender Conformity × Age Group × Participant Gender	0.60	.440	.00
Peer Gender × Peer Gender Conformity × Task Order × Participant Gender	2.47	.118	.01
Peer Gender × Peer Gender Conformity × Age Group × Task Order	0.04	.844	.00
Peer Gender × Peer Gender Conformity × Age Group × Task Order × Participant Gender	0.00	.969	.00
Peer Gender × Trait Type	0.44	.510	.00
Peer Gender × Trait Type × Participant Gender	0.84	.360	.00
Peer Gender × Trait Type × Age Group	0.11	.745	.00
Peer Gender × Trait Type × Task Order	3.00	.085	.01
Peer Gender × Trait Type × Age Group × Participant Gender	0.03	.855	.00
Peer Gender × Trait Type × Task Order × Participant Gender	0.42	.519	.00
Peer Gender × Trait Type × Age Group × Task Order	0.11	.747	.00
Peer Gender × Trait Type × Age Group × Task Order × Participant Gender	0.38	.536	.00
Peer Gender Conformity × Trait Type	0.66	.419	.00
Peer Gender Conformity × Trait Type × Participant Gender	3.58	.060	.02
Peer Gender Conformity × Trait Type × Age Group	2.15	.144	.01
Peer Gender Conformity × Trait Type × Task Order	0.04	.837	.00
Peer Gender Conformity × Trait Type × Age Group × Participant Gender	0.32	.570	.00
Peer Gender Conformity × Trait Type × Task Order × Participant Gender	1.37	.243	.01
Peer Gender Conformity × Trait Type × Age Group × Task Order	0.80	.372	.00
Peer Gender Conformity × Trait Type × Age Group × Task Order × Participant Gender	0.03	.875	.00
Peer Gender × Peer Gender Conformity × Trait Type	1.62	.204	.01
Peer Gender × Peer Gender Conformity × Trait Type × Participant Gender	0.03	.872	.00
Peer Gender × Peer Gender Conformity × Trait Type × Age Group	1.55	.214	.01
Peer Gender × Peer Gender Conformity × Trait Type × Task Order	0.02	.876	.00
Peer Gender × Peer Gender Conformity × Trait Type × Age Group × Participant Gender	2.89	.091	.01
Peer Gender × Peer Gender Conformity × Trait Type × Task Order × Participant Gender	0.13	.718	.00
Peer Gender × Peer Gender Conformity × Trait Type × Age Group × Task Order	0.26	.613	.00
Peer Gender × Peer Gender Conformity × Trait Type × Age Group × Task Order × Participant Gender	0.06	.810	.00

Section S9: References

JASP Team. (2026). JASP (Version 0.98.1) [Computer software]. https://jasp-stats.org/

Jeffreys, H. (1998). *The theory of probability* (3rd ed.). Oxford University Press.

Rouder, J. N., Speckman, P. L., Sun, D., Morey, R. D., & Iverson, G. (2009). Bayesian *t* tests for accepting and rejecting the null hypothesis. *Psychonomic Bulletin & Review*, *16*(2), 225–237. https://doi.org/10.3758/PBR.16.2.225

Wagenmakers, E. J., Marsman, M., Jamil, T., Ly, A., Verhagen, J., Love, J., Selker, R., Gronau, Q. F., Šmíra, M., Epskamp, S., Matzke, D., Rouder, J. N., & Morey, R. D. (2018). Bayesian inference for psychology. Part I: Theoretical advantages and practical ramifications. *Psychonomic Bulletin & Review*, *25*(1), 35–57. https://doi.org/10.3758/s13423-017-1343-3

Wetzels, R., Matzke, D., Lee, M. D., Rouder, J. N., Iverson, G. J., & Wagenmakers, E. J. (2011). Statistical evidence in experimental psychology: An empirical comparison using 855 *t* tests. *Perspectives on Psychological Science*, *6*(3), 291–298. https://doi.org/10.1177/1745691611406923
